# Identification of mitophagy-related biomarkers and immune infiltration in major depressive disorder

**DOI:** 10.1186/s12864-023-09304-6

**Published:** 2023-04-25

**Authors:** Jing Zhang, Shujun Xie, Rong Xiao, Dongrong Yang, Zhi Zhan, Yan Li

**Affiliations:** 1 The Second Clinical Medical College, Guangzhou University of Chinese Medicine, Guangzhou, 510405 China; 2 Department of Hematology and Oncology, The Third Affiliated Hospital of Guangzhou University of Chinese Medicine, Guangzhou, 510378 China; 3 Department of Rehabilitation, The Eighth People’s Hospital of Hefei, Hefei, 238000 China; 4 Department of Psychological Sleep, Guangdong Provincial Hospital of Chinese Medicine, Guangzhou, 510120 China

**Keywords:** Major depressive disorder, Mitophagy, Immune infiltration, Mitophagy-related gene, Gene expression omnibus database, Bioinformatic analysis

## Abstract

**Background:**

Major depressive disorder (MDD) is a life-threatening and debilitating mental health condition. Mitophagy, a form of selective autophagy that eliminates dysfunctional mitochondria, is associated with depression. However, studies on the relationship between mitophagy-related genes (MRGs) and MDD are scarce. This study aimed to identify potential mitophagy-related biomarkers for MDD and characterize the underlying molecular mechanisms.

**Methods:**

The gene expression profiles of 144 MDD samples and 72 normal controls were retrieved from the Gene Expression Omnibus database, and the MRGs were extracted from the GeneCards database. Consensus clustering was used to determine MDD clusters. Immune cell infiltration was evaluated using CIBERSORT. Functional enrichment analyses were performed to determine the biological significance of mitophagy-related differentially expressed genes (MR-DEGs). Weighted gene co-expression network analysis, along with a network of protein–protein interactions (PPI), was used to identify key modules and hub genes. Based on the least absolute shrinkage and selection operator analysis and univariate Cox regression analysis, a diagnostic model was constructed and evaluated using receiver operating characteristic curves and validated with training data and external validation data. We reclassified MDD into two molecular subtypes according to biomarkers and evaluated their expression levels.

**Results:**

In total, 315 MDD-related MR-DEGs were identified. Functional enrichment analyses revealed that MR-DEGs were mainly enriched in mitophagy-related biological processes and multiple neurodegenerative disease pathways. Two distinct clusters with diverse immune infiltration characteristics were identified in the 144 MDD samples. *MATR3*, *ACTL6A*, *FUS*, *BIRC2*, and *RIPK1* have been identified as potential biomarkers of MDD. All biomarkers showed varying degrees of correlation with immune cells. In addition, two molecular subtypes with distinct mitophagy gene signatures were identified.

**Conclusions:**

We identified a novel five-MRG gene signature that has excellent diagnostic performance and identified an association between MRGs and the immune microenvironment in MDD.

**Supplementary Information:**

The online version contains supplementary material available at 10.1186/s12864-023-09304-6.

## Introduction

Major depressive disorder (MDD) is a heterogeneous, recurrent, and life-threatening mental disorder characterized by depressed mood, self-accusation, self-guilt, anhedonia, and suicidality, leading to a significant decrease in overall quality of life [1, 2]. MDD has been identified as the main risk factor for death by suicide [3] and the main cause of disability [4], presenting a substantial economic and social burden worldwide. Due to the lack of reliable biomarkers, the diagnosis of MDD mainly relies on the symptoms of patients and depression rating scales, resulting in a high rate of misdiagnosis [5]. Although a wide variety of antidepressants are available, 30–50% of patients with MDD do not achieve complete remission [6], reflecting that the conventional therapies do not address the important biological processes involved in MDD pathology. Therefore, a better understanding of the underlying pathophysiological mechanisms of MDD is necessary to identify possible treatment targets for the development of biomarkers that help provide a more accurate and early diagnosis.

Mitochondria are crucial organelles in eukaryotic cells and are key regulators of physiological processes such as adenosine triphosphate (ATP) synthesis, reactive oxygen species generation and scavenging, and apoptosis in the cell life cycle. Selective mitochondrial autophagy, also known as mitophagy, is an important mitochondrial quality control mechanism that eliminates damaged mitochondria [7, 8]. Several studies have reported that mitophagy and subsequent mitochondrial dysfunction are significant contributors to the pathophysiology of MDD [9–11]. Mitophagy has also been considered the emerging mechanism of action for some antidepressants [12, 13]. Shu et al. discovered that the antidepressant fluoxetine protects astrocytes by enhancing astrocytic mitophagy and removing damaged mitochondria in a corticosteroid-treated cell model [12]. However, mitophagy-related biomarkers in MDD have not yet been fully elucidated, although mitophagy plays an important role in MDD. The identification of mitophagy-related genes (MRGs) associated with MDD is therefore urgently needed so that new biomarkers and therapeutic targets can be developed.

Several studies have shown that immune dysregulation and activation of the inflammatory response system (IRS) are associated with the pathogenesis of MDD [14–16]. Of note, recent research has shown the vital importance of mitophagy in controlling the secretion of inflammatory cytokines and the homeostasis and differentiation of immune cells, which are relevant to the pathogenesis of inflammatory and autoimmune diseases [17, 18]. Interestingly, it has been reported that the severity of MDD may be affected by the crosstalk between mitophagy and inflammation [9]. Although the respective roles of mitophagy and immunity in MDD have been reported [10, 16], the interaction between mitophagy and immune infiltration that affects the MDD process is unclear and requires further investigation.

In this study, the biological significance of MRGs and their relationship with immune infiltration in MDD and MDD subtypes were analyzed. First, we retrieved MDD-related genes and MRGs from the Gene Expression Omnibus (GEO) and GeneCards databases, respectively. Subsequently, we identified MDD subclusters using consensus clustering based on 144 MDD samples. We performed multiple functional enrichment analyses to better understand the biological significance of MRGs in MDD. MDD mitophagy-related biomarkers were obtained by weighted gene co-expression network analysis (WGCNA), least absolute shrinkage and selection operator (LASSO) logistic regression, and receiver operating characteristic (ROC) curve analysis. Using the CIBERSORT algorithm, we compared the immune microenvironment of patients with MDD and controls, as well as MDD subclusters, to evaluate the molecular immunological mechanisms underlying the development of MDD. The correlation between the diagnostic markers and immune cells was also examined. Overall, the results of this study may contribute to an improved understanding of the pathophysiology of MDD at the molecular level and may identify novel biomarkers for its diagnosis.

## Materials and methods

### Data pretreatment

Two gene expression profile datasets for MDD samples, GSE32280 [19] and GSE98793 [20], were obtained from the GEO database. GSE32280 contains 16 MDD cases and eight normal controls, while GSE98793 contains 128 patients with MDD and 64 healthy controls. Both datasets were derived from the GPL570 platform (Affymetrix Human Genome U133 Plus 2.0 Array). We merged and batched the normalized GSE32280 and GSE98793 datasets into the training group using the R package “sva” [21]. After consolidating the data, a final sample of 144 patients with MDD and 72 healthy controls remained. The diagnostic validation set GSE190518 [22] consisted of four MDD samples and four normal controls, all of which were derived from the GPL20301 platform (Illumina HiSeq 4000, Homo sapiens). Supplementary Table S1 presents details of the datasets.

To explore the importance of mitophagy in MDD and the expression of MRGs in MDD samples, we retrieved 2414 MRGs with a relevance score > 1 from the GeneCards database [23] searched using the keyword “Mitophagy.”

### Immune cell infiltrate analysis

The CIBERSORT algorithm [24] was used to assess the proportion of infiltrating immune cells in 144 MDD samples and 72 normal controls, given the importance of immune infiltration cells in the progression of MDD. Wilcoxon rank-sum tests were performed to evaluate the differences between MDD samples and normal controls regarding the proportion of infiltrating immune cells [25, 26]. Furthermore, the correlations between 22 immune cell subpopulations in MDD samples and controls were evaluated separately using Pearson’s correlation analysis and visualized using the “ggplot2” R package. Statistical significance was defined as *p*-value < 0.05 and correlation coefficient (r) > 0.3.

### Consensus clustering analysis for MDD

The ConsensusClusterPlus package [27] was used to perform consensus cluster analysis on the 144 standardized MDD samples to detect possible heterogeneity and identify subgroups within MDD. Using the R package “factoextra,” we determined the optimal number of clusters and then used the k-means algorithm to cluster unsupervised MDD samples.

### Identification of differentially expressed genes among MDD clusters

Differentially expressed genes (DEGs) among MDD clusters were identified using the “limma” package in R [28]. Genes with *p* < 0.05 and |Log2FC (fold-change) |> 1.5 were considered DEGs for further analysis. The heatmap and volcano plot of the DEGs were generated using the “heatmap” and “ggplot2” R packages, respectively. Additionally, to investigate the expression profile of MRGs between MDD subgroups, mitophagy-related differentially expressed genes (MR-DEGs) were identified by the intersection of DEGs and MRGs.

### Analysis of functional enrichment

Multiple functional enrichment analyses were performed to better understand the molecular mechanisms and signaling cascades underlying the involvement of MR-DEGs in MDD. Using the “clusterProfiler” [29] and “DOSE” [30] R packages, we performed Gene Ontology (GO), Kyoto Encyclopedia of Genes and Genomes (KEGG) pathway [31–33], and Disease Ontology (DO) enrichment analyses of MR-DEGs. The enrichment outcomes with an adjusted *p* < 0.05 were selected for presentation as a bubble plot.

To better understand the biological and pathway differences between the two gene sets, we performed gene set enrichment (GSEA) and gene set variation (GSVA) analyses. We downloaded the reference gene sets of “c5.go. v7.5.1. entrez.gmt” and “c2.cp.kegg. v7.5.1. entrez.gmt” for GSEA from the Molecular Signatures database [34]. Statistical significance was set at *p* < 0.05. To evaluate the differences in functional enrichment between the disease and control groups, GSVA was performed using the R package “GSVA.”

### Identifying key modules in the co-expression network

To identify important modules and key genes among clusters of MDD, we performed WGCNA analysis using the “WGCNA” package in R [35]. First, 144 MDD samples were clustered to remove outliers. Second, an optimal soft threshold parameter beta was adopted to construct scale-free co-expression networks. Subsequently, gene modules were identified using a dynamic tree-cutting algorithm based on the topological overlap measure, and gene modules were identified. Modules with a minimum module size of 30 and a cut height of 0.2 were merged. The correlations between the clusters and gene modules were calculated and visualized using a heatmap. Modules with the highest correlations were identified and analyzed.

### Establishment of Protein–protein Interaction (PPI) networks and screening of hub genes

A Venn diagram package in R was used to identify overlapping genes associated with mitophagy and MDD by taking the intersection of MRGs, DEGs, and module genes obtained by WGCNA. Subsequently, to systematically analyze the biological functions of the overlapping genes, the genes were mapped to the STRING database, which is a tool that predicts interactions between genes or proteins [36]. A PPI network with a combined score greater than 0.4 was reserved and the results were imported to Cytoscape (version 3.7.2) [37] for advanced analysis. We then used Cytoscape’s plug-in cytoHubba [38] and the maximal clique centrality (MCC) algorithm to extract the 10 most significant hub genes in the PPI network.

### Construction and validation of the diagnostic model

To identify candidate genes that serve as biomarkers differentiating MDD and normal samples, we first used the combined microarray datasets (GSE32280 and GSE98793) as a training set (containing 144 MDD samples and 72 control samples) and then validated them using an independent dataset (GSE190518, containing four MDD samples and 4 control samples).

Based on the aforementioned training dataset, we used the “glmnet” R package for LASSO and performed the univariate Cox regression analyses to identify key mitophagy-related biomarkers. “Forest plot” packages were used to visualize the Cox results. For further calculation of the risk score, genes with nonzero coefficients in the LASSO regression model were selected. We computed the risk score for each included gene using the following formula:

$$\mathrm{riskScore}={\textstyle\sum_{\mathrm i}}\mathrm{Coefficient}\;\left({\mathrm{gene}}_{\mathrm i}\right)\;\ast\;\mathrm{mRNA}\;\mathrm{Expression}\;({\mathrm{gene}}_{\mathrm i}).$$

The efficacy of the risk score system for predicting MDD was then evaluated by receiver operating characteristic (ROC) curve analysis using the R package “pROC” [39].

To further evaluate the diagnostic performance and robustness of candidate biomarkers, the GSE190518 dataset was used as a validation set. Furthermore, the area under the curve (AUC) for candidate biomarkers was computed using the “pROC” in the R package to assess their diagnostic value. AUC > 0.5 and *P* < 0.05 were used as diagnostic criteria.

### Analysis of immune infiltrating cells in MDD clusters

Using the CIBERSORT algorithm, we analyzed immune cell infiltration between different clusters of MDD samples. The correlation between immune cells and the expression of the 10 hub genes was further examined in subsequent analyses. R package “ggplot2” was used to visualize the results of the correlation analysis. In addition, the correlations between the immune cell subsets in the two clusters were evaluated separately.

### Identification of MDD subtypes based on biomarkers

Based on the expression of biomarker characteristics, MDD was divided into different subtypes using the ConsensusCluster Plus package. Differences between subtypes were visualized using principal component analysis (PCA). We used LASSO and univariate Cox regression analysis to investigate the association between biomarkers and MDD subtypes. We also used Pearson’s correlation analysis to determine which biomarkers were correlated with each other. Subsequently, the expression levels of the 10 hub genes in clusters and subtypes were compared to determine whether both types of classification were consistent.

### Statistical methods

R version 4.0.2 was used for all data analyses. To compare the two sets of continuous variables, we used the Student’s t-test for variables with a normally distributed distribution and the Mann–Whitney U test for variables with atypical distributions. Pearson’s correlation coefficient was used to assess the relationship between the variables (R version 4.0.2). Multiple test correction (Bonferroni correction) was used to adjust the *p*-values. Statistical significance was defined as *p* < 0.05, and all *p*-values were two-sided.

## Results

### Data preprocessing

**Fig. 1:**
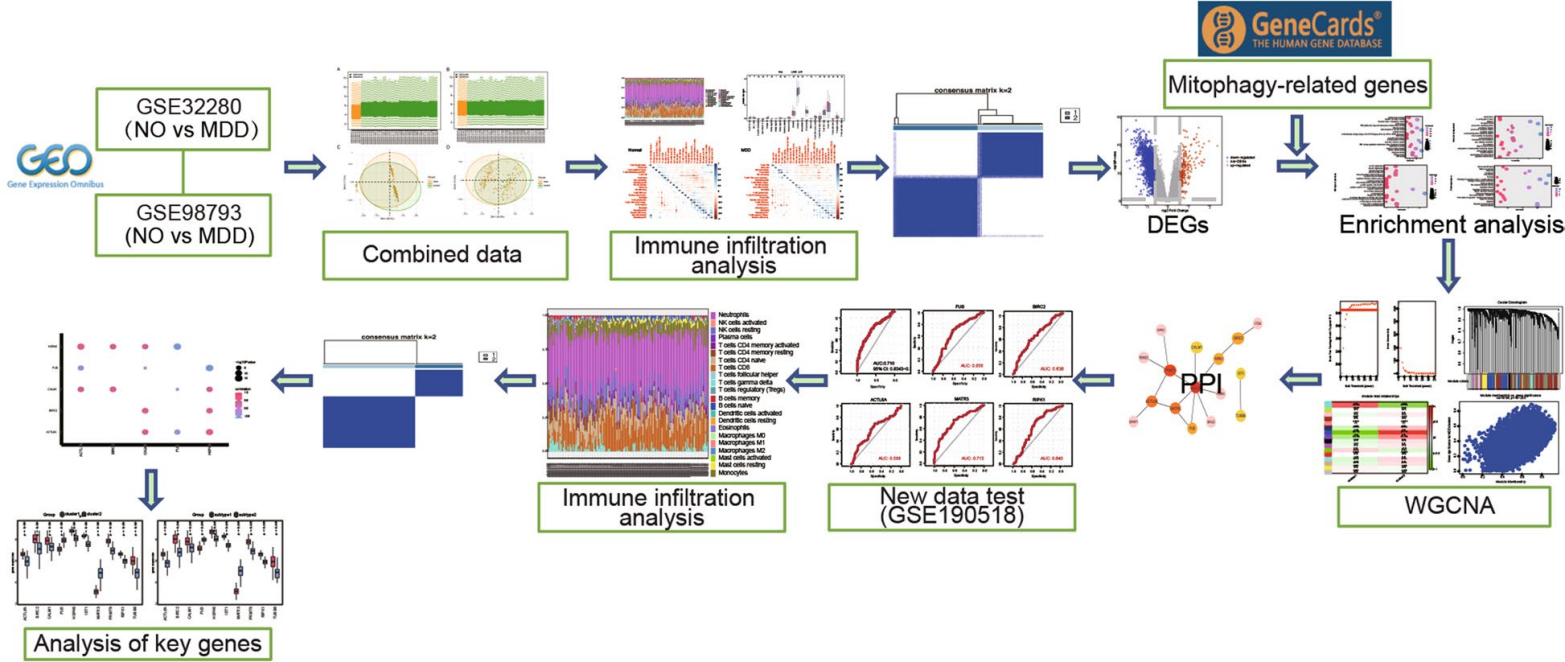
The flowchart of the study

**Fig. 2:**
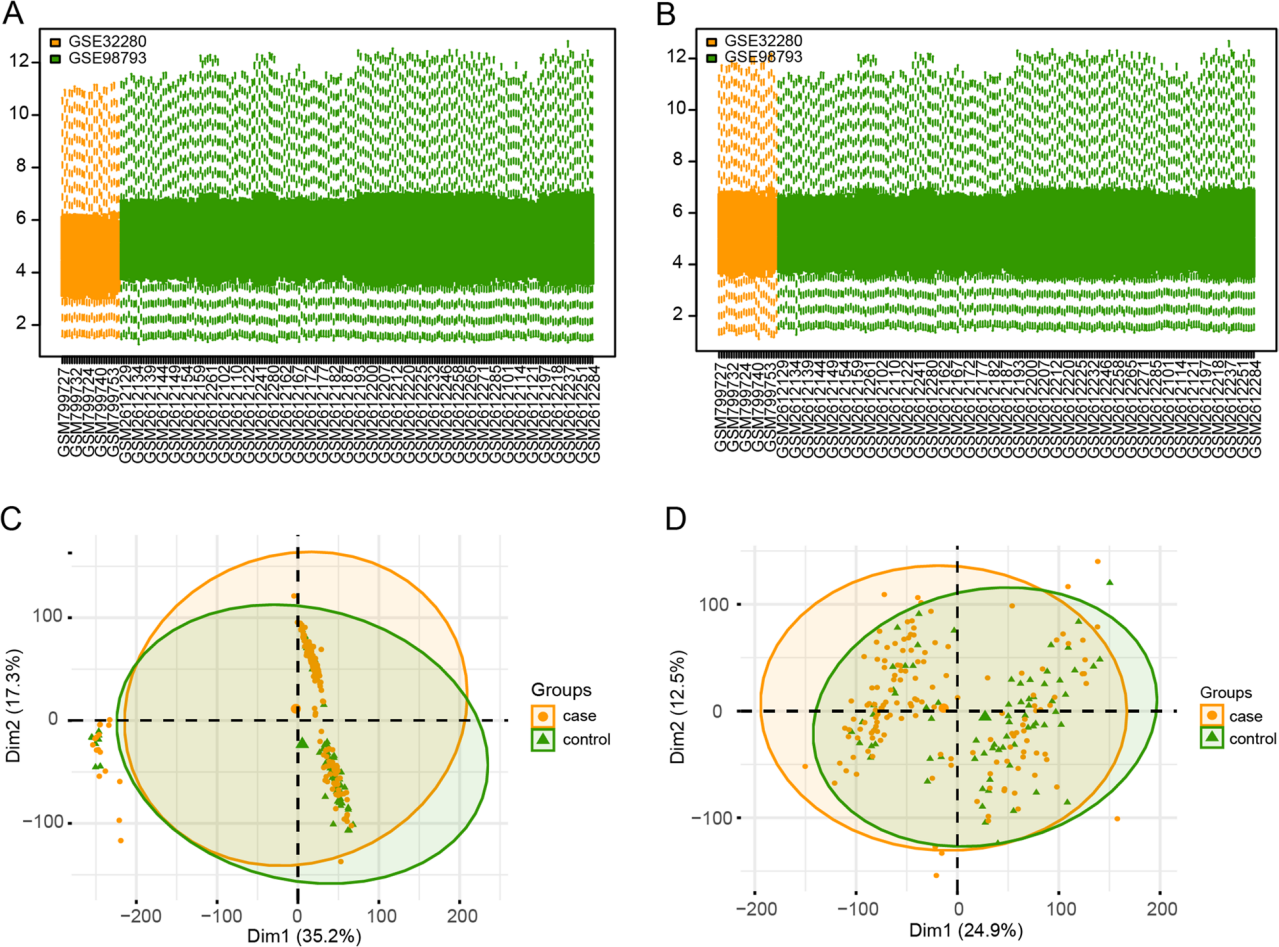
Data preprocessing. **A** and **B** The boxplot of the merged microarray datasets before and after normalization. **C** and **D** Two scatterplots depicting principal component analysis of normalized gene expression data before and after the removal of batch effects

Figure 1 shows the flowchart of the study. The integrated gene expression profile was obtained after eliminating batch effects between the two datasets (GSE32280 and GSE98793), which contained 144 MDD samples and 72 normal controls, and 21,755 genes were identified. As shown in Fig. 2, the normalized boxplots of gene expression profiles showed differences before and after standardization pretreatment. As a consequence of normalization and batch-effect adjustment, the expression distributions of all samples are more consistent, making the downstream analysis more accurate and robust.

### Immune infiltration analysis

**Fig. 3:**
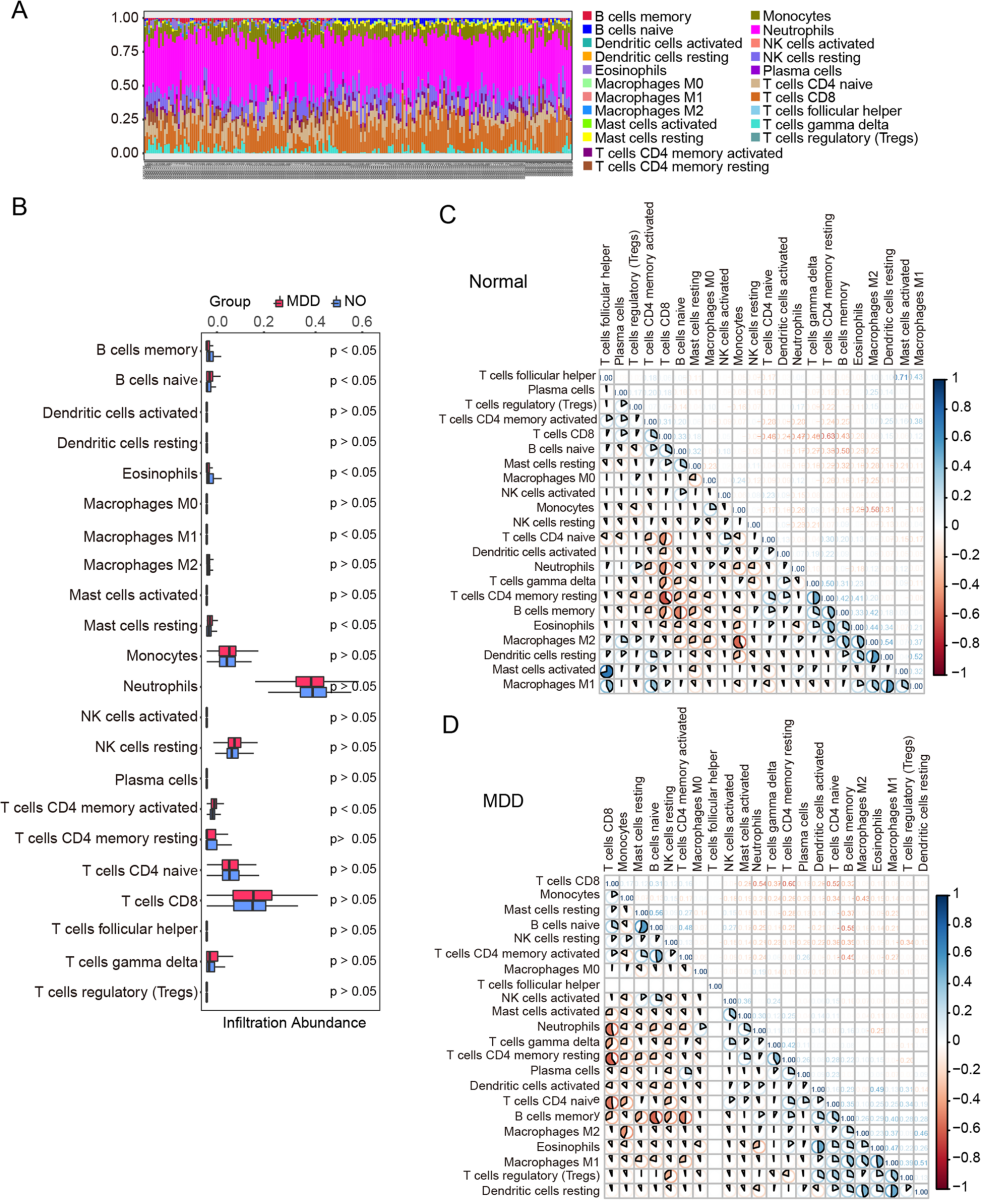
Immune infiltration analysis. **A** Analysis of 22 types of immune cells in major depressive disorder (MDD) samples and normal controls. **B** Differences in immune infiltration abundance between normal controls and MDD. The blue color indicates normal controls, and the red color indicates MDD samples. **C** The matrix of correlations between immune cells in normal samples. **D** The matrix of correlations between immune cells in MDD samples. Blue color indicates a positive correlation and red color indicates a negative correlation

An in-depth analysis of immune infiltration between MDD samples and normal controls was performed using the CIBERSORT algorithm (Fig. 3). First, we represent a stacking graph of the fraction of immune cells at each level in 144 patients with MDD and 72 control samples. A significant difference in the proportion of immune cells was observed between these samples, indicating that MDD samples should be re-clustered (Fig. 3A). Compared to normal controls, MDD samples tended to have higher proportions of naïve B cells, M1 macrophages, resting mast cells, and activated memory CD4 + T cells and lower proportions of memory B cells and eosinophils (Fig. 3B). Correlations between the immune cell subpopulations in MDD and the control samples were determined separately. Activated mast cells were positively correlated with T follicular helper cells in normal controls, while M2 macrophages were negatively correlated with monocytes (Fig. 3C). In contrast, in MDD samples, a negative correlation was detected between CD8 + T cells and several other types of immune cells, including activated natural killer (NK) cells, activated mast cells, neutrophils, resting memory CD4 + T cells, and gamma delta T cells (Fig. 3D). Considering the data mentioned above, it can be shown from different perspectives that the immunological microenvironment of MDD samples differs from that of normal samples.

### Identification of MDD clusters and DEGs

**Fig. 4:**
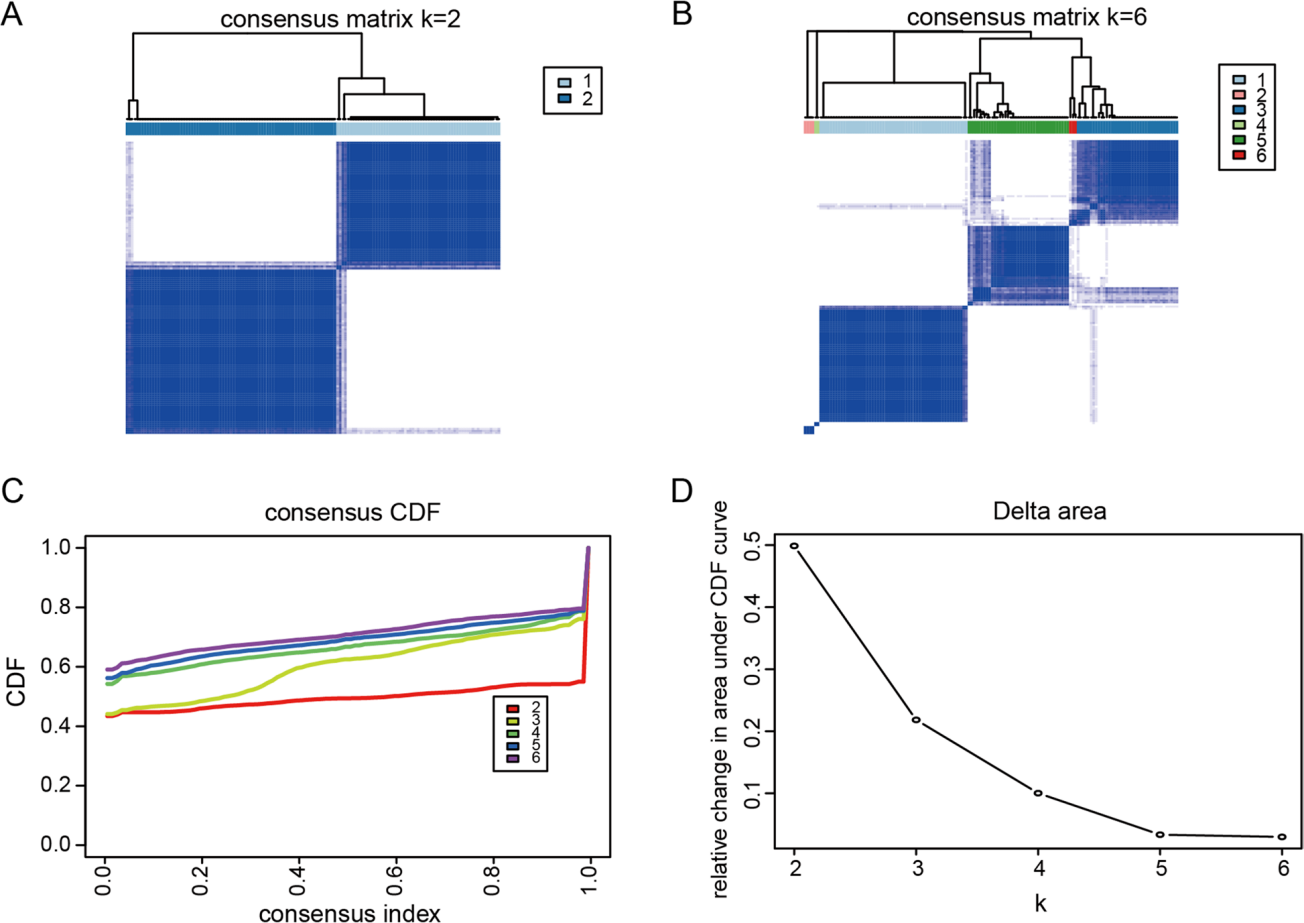
Consensus clustering of 144 major depressive disorder (MDD) samples. **A**-**B** Matrix of consensus clustering for 144 MDD samples from k = 2 to k = 6. **C** Cumulative distribution function (CDF) is calculated based on consensus for k = 2 to k = 6. (D) The area under the CDF curve for k = 2–6 represents a relative change

We categorized 144 MDD samples into two clusters using a consensus clustering analysis. As shown in Fig. 4, k = 2 was considered the optimal number of clusters based on the relative change in the area under the cumulative distribution function (CDF) curve. Consequently, two clusters of MDD were identified and labeled as cluster 1 and cluster 2 (cluster 1: 63 samples and cluster 2: 81 samples).

**Fig. 5:**
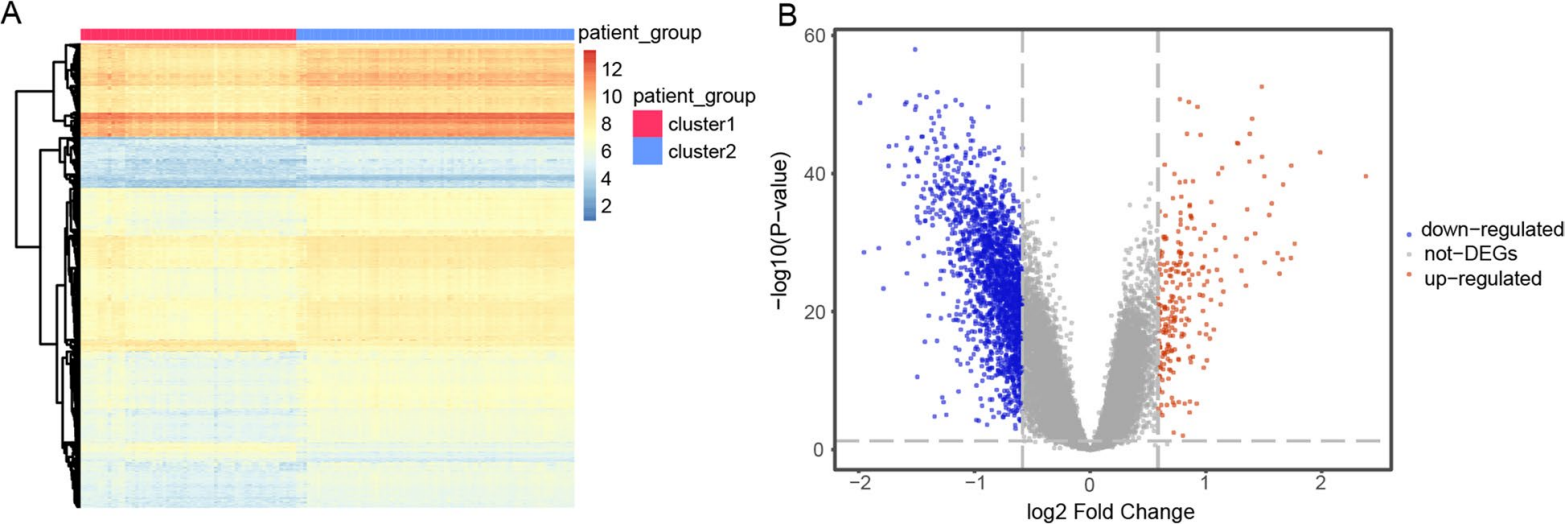
Differential expression analysis between two clusters of major depressive disorder (MDD). **A** Heatmap results of differentially expressed genes (DEGs). The red color represents cluster 1 and blue color represents cluster 2. **B** Volcano plot of DEGs

Additionally, to further explore the heterogeneity between the two clusters, we obtained 2,059 DEGs with *p* < 0.05 and *p* > 1.5 for |log FC (fold-change) |. A heatmap of 2,059 DEGs is shown in Fig. 5A. There were 241 upregulated DEGs and 1,818 downregulated DEGs in cluster 1 compared to those in cluster 2 (Fig. 5B).

### Functional enrichment analysis

**Fig. 6:**
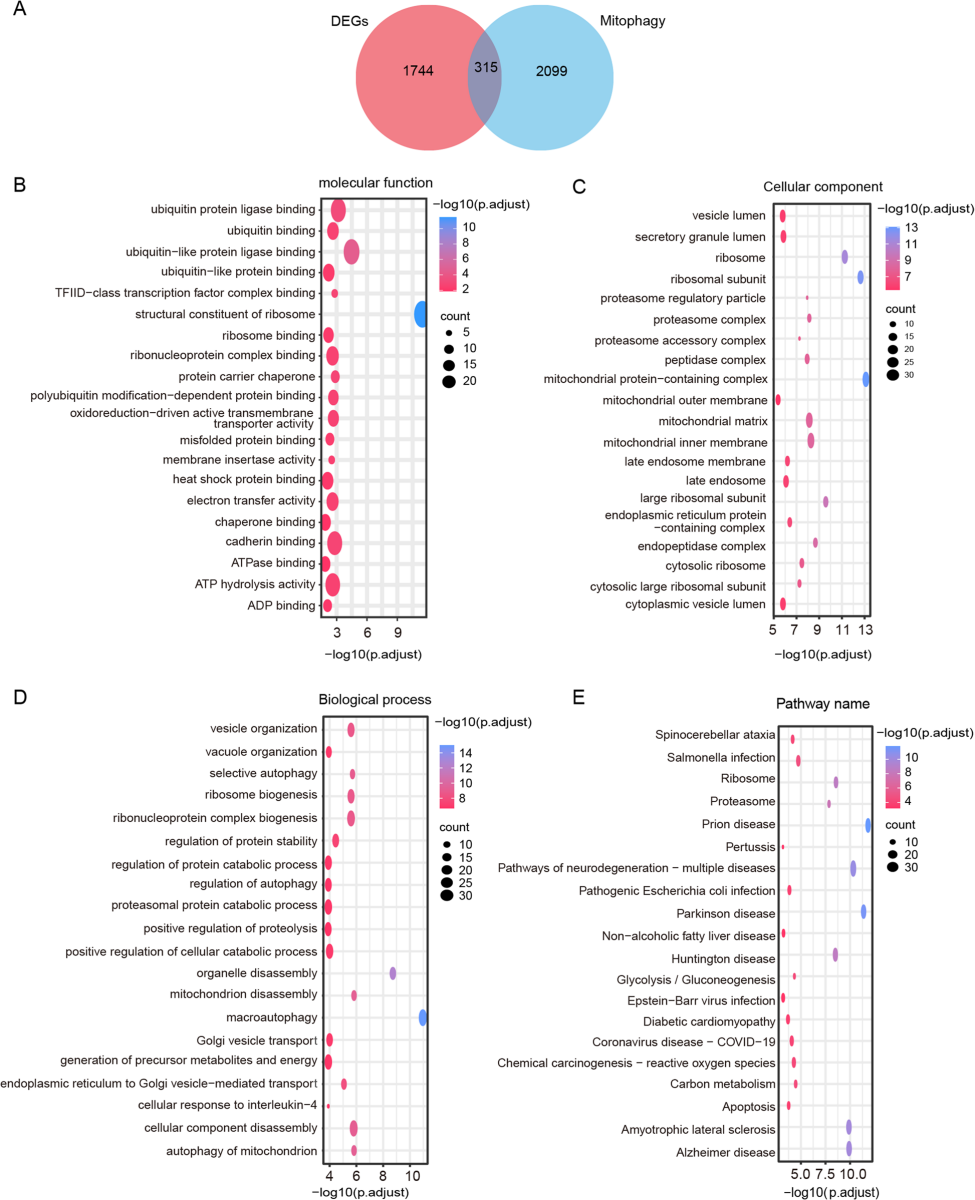
An analysis of the functional enrichment of MR-DEGs. **A** The intersection of MRGs and DEGs is shown in a Venn diagram. **B**-**D** Twenty of the most significant gene ontology terms. **E** Twenty of the most significant Kyoto Encyclopedia of Genes and Genomes pathways

To investigate the biological functions of MRGs in MDD clusters in depth, we performed a functional enrichment analysis of MR-DEGs. First, we identified 315 MR-DEGs from the 2,059 DEGs intersecting with the 2,414 MRGs (Fig. 6A). Subsequently, 315 MR-DEGs linked to MDD were analyzed using GO and KEGG analyses to understand their biological functions and signaling pathways (Supplementary Table S2). The results of GO enrichment showed that in biological processes (BP), MR-DEGs were considerably enriched in macroautophagy, organelle disassembly, mitochondrial autophagy, mitochondrial disassembly, and cellular component disassembly. In the cell component (CC), MR-DEGs were abundant primarily in mitochondrial protein-containing complexes, ribosomal subunits, ribosomes, large ribosomal subunits, and endopeptidase complexes. Regarding molecular function, these genes play an essential role in several key functions, such as being the structural constituent of the ribosome, ubiquitin-like protein ligase binding, and protein carrier chaperone (Fig. 6B-D). KEGG pathway enrichment analyses revealed that MR-DEGs were significantly enriched in pathways relevant to Parkinson’s disease (PD), Alzheimer’s disease (AD), amyotrophic lateral sclerosis (ALS), and multiple neurodegenerative diseases (Fig. 6E).

**Fig. 7:**
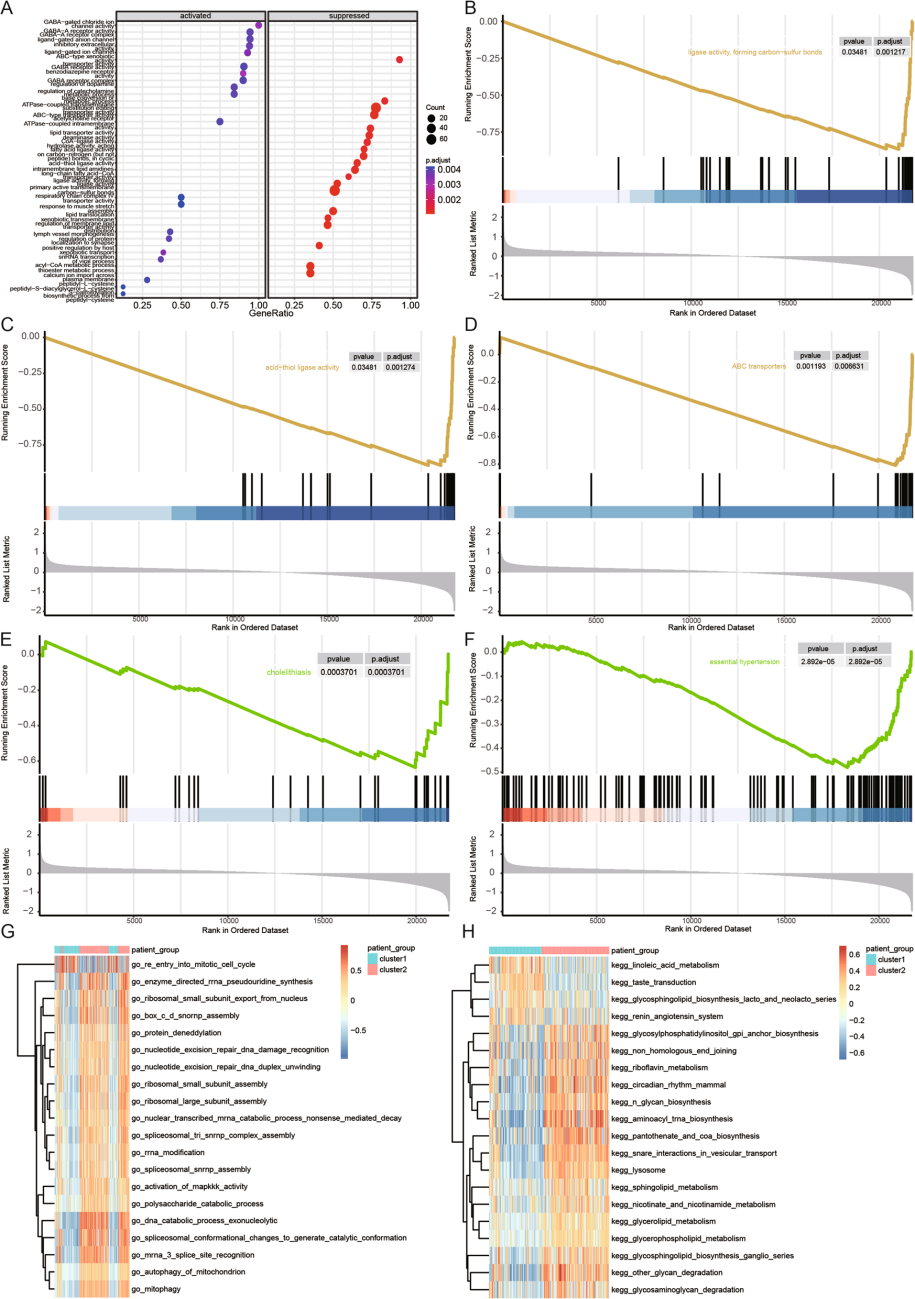
Visualization of the results of GSEA and GSVA analyses. **A** Analysis of gene ontology (GO) functional enrichment. **B**-**C** The results of gene set enrichment analysis (GSEA) (GO terms). **D** The results of GSEA (Kyoto Encyclopedia of Genes and Genomes [KEGG] pathways). **E**–**F** The results of GSEA (disease ontology pathways). **G** The results of gene set variation analysis (GSVA) (GO terms). **H** The results of GSVA (KEGG pathways). Clusters 1 and 2 are represented by blue and red colors, respectively

To investigate the underlying functional differences between the two clusters of MDD, we performed a GSEA analysis (Supplementary Table S3). Based on GO term gene sets, GSEA revealed that ligand-gated anion channel activity, GABA receptor activity, and GABA-A receptor complex were noticeably upregulated in cluster1 (Fig. 7A), while biological processes, such as ligase activity, forming carbon–sulfur bonds, and acid-thiol ligase activity, were noticeably downregulated (Fig. 7B, C). Meanwhile, KEGG enrichment results by GSEA showed that nicotine addiction was noticeably upregulated in cluster 1, while pathways, such as ABC transporters and collecting duct acid secretion, were noticeably downregulated (Fig. 7D). In particular, consistent with the above KEGG analysis, the DO analysis revealed that the genes involved in the two clusters were associated with ALS, essential hypertension, and cholelithiasis (Fig. 7E, F, Supplementary Table S4).

According to GSVA, the results of GO terms showed that enzyme-directed rRNA pseudouridine synthesis and ribosomal small subunit export from the nucleus were activated in cluster 2 samples, while terms, such as re-entry into the mitotic cell cycle, were suppressed (Fig. 7G, Supplementary Table S5-1). KEGG enrichment results by GSVA showed that glycosylphosphatidylinositol gpi anchor biosynthesis, riboflavin metabolism, and non-homologous end joining were noticeably activated in cluster 2, while pathways, such as linoleic acid metabolism and taste transduction, were suppressed (Fig. 7H, Supplementary Table S5-2). Overall, the results of GSEA and GSVA indicated significant differences in biological processes and pathways between cluster 1 and cluster 2.

### Establishment of a co-expression network and identification of core modules

**Fig. 8:**
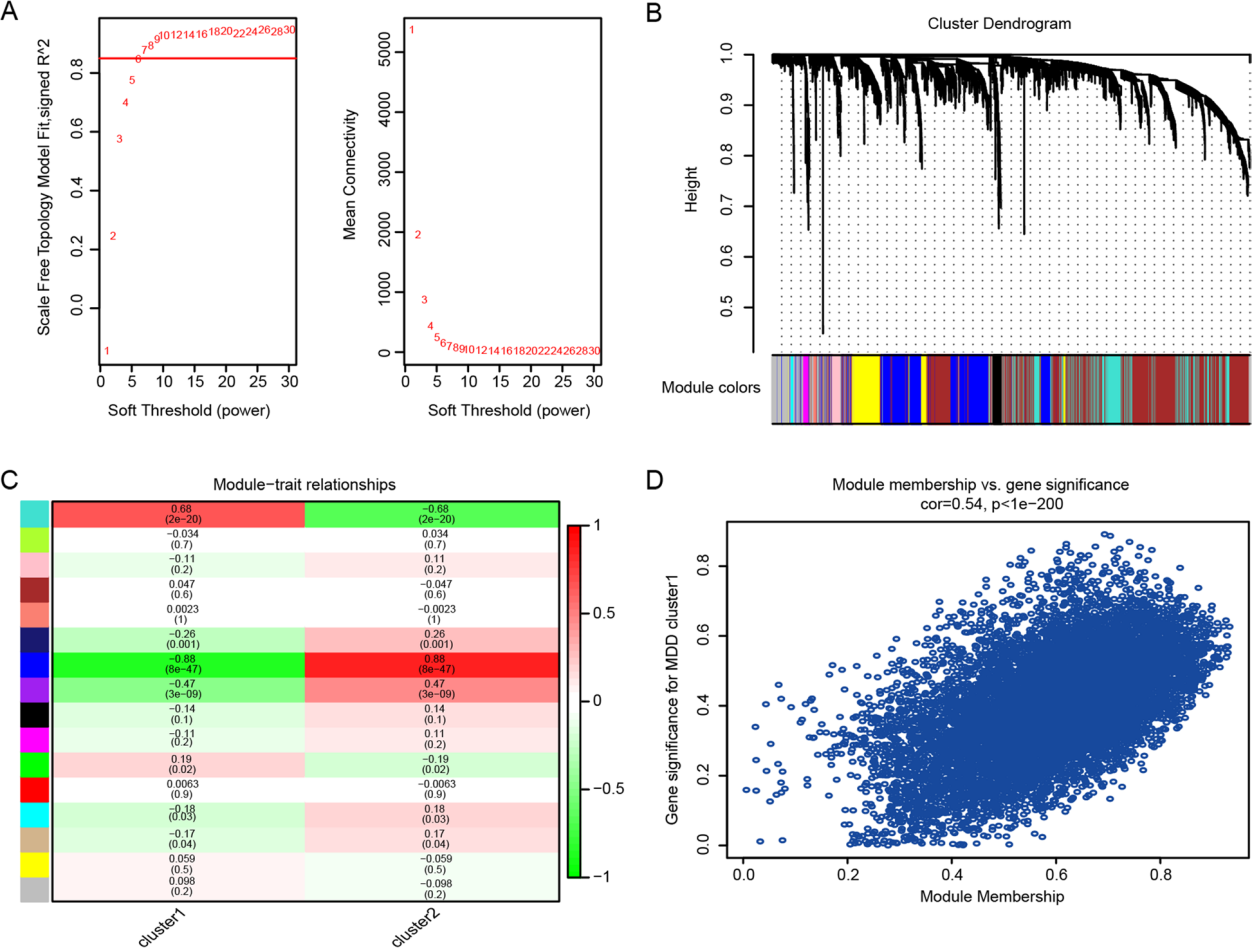
Weighted gene co-expression network analysis (WGCNA). **A** An analysis of the scale-free fit index and the mean connectivity for selected soft threshold powers (β). **B** WGCNA’s Hierarchical Cluster Tree reveals co-expression modules. **C** Heatmap showing the relationship between gene modules and clusters. **D** Module eigengene scatter plot for the blue module

WGCNA was used to identify co-expression modules to determine which genes were functionally related to MDD clusters. By setting the soft threshold power to six (scale-free R ^2^ = 0.85), 16 modules were identified (Fig. 8A, B). As shown in Fig. 8C, module-trait association analysis revealed a significant positive correlation between the blue module and cluster 1. Figure 8D shows a scatter plot of GS versus module membership for the blue module (Cor = 0.54, *p* < 1e-200). In total, 9,395 genes were identified for further analysis using the blue module.

### Network analysis of PPIs and identification of hub genes

**Fig. 9:**
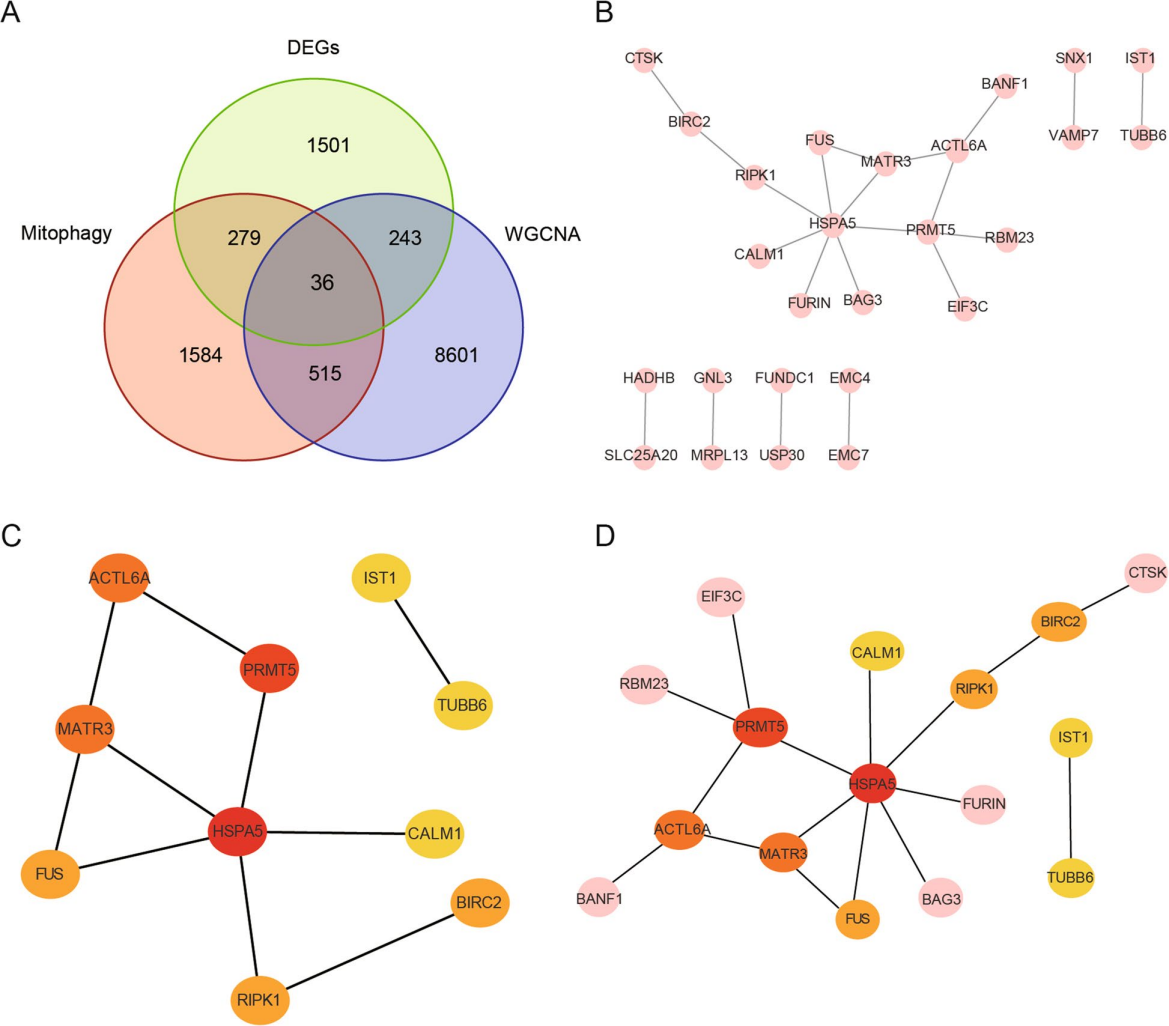
Analysis of protein–protein interaction (PPI) networks and identification of hub genes. **A** Venn diagram of the overlapping genes. **B** PPI network of 36 overlapping targets. **C**, **D** Top 10 hub genes identified using maximal clique centrality (MCC) and cytohubba. A higher MCC value is associated with a darker color

By intersecting 2,059 DEGs, 2,414 MRGs, and 9,395 module genes, we identified 36 overlapping genes related to mitophagy and MDD (Fig. 9A). PPI analysis of the 36 overlapping genes was performed using the STRING database and visualized using Cytoscape V3.9.0 (Fig. 9B). The MCC approach using the cytoHubba plug-in selected *HSPA5*, *PRMT5*, *MATR3*, *ACTL6A*, *FUS*, *BIRC2*, *RIPK1*, *IST1*, *TUBB6*, and *CALM1* as the top 10 hub genes (Fig. 9C, D).

### Construction and validation of diagnostic models

**Fig. 10:**
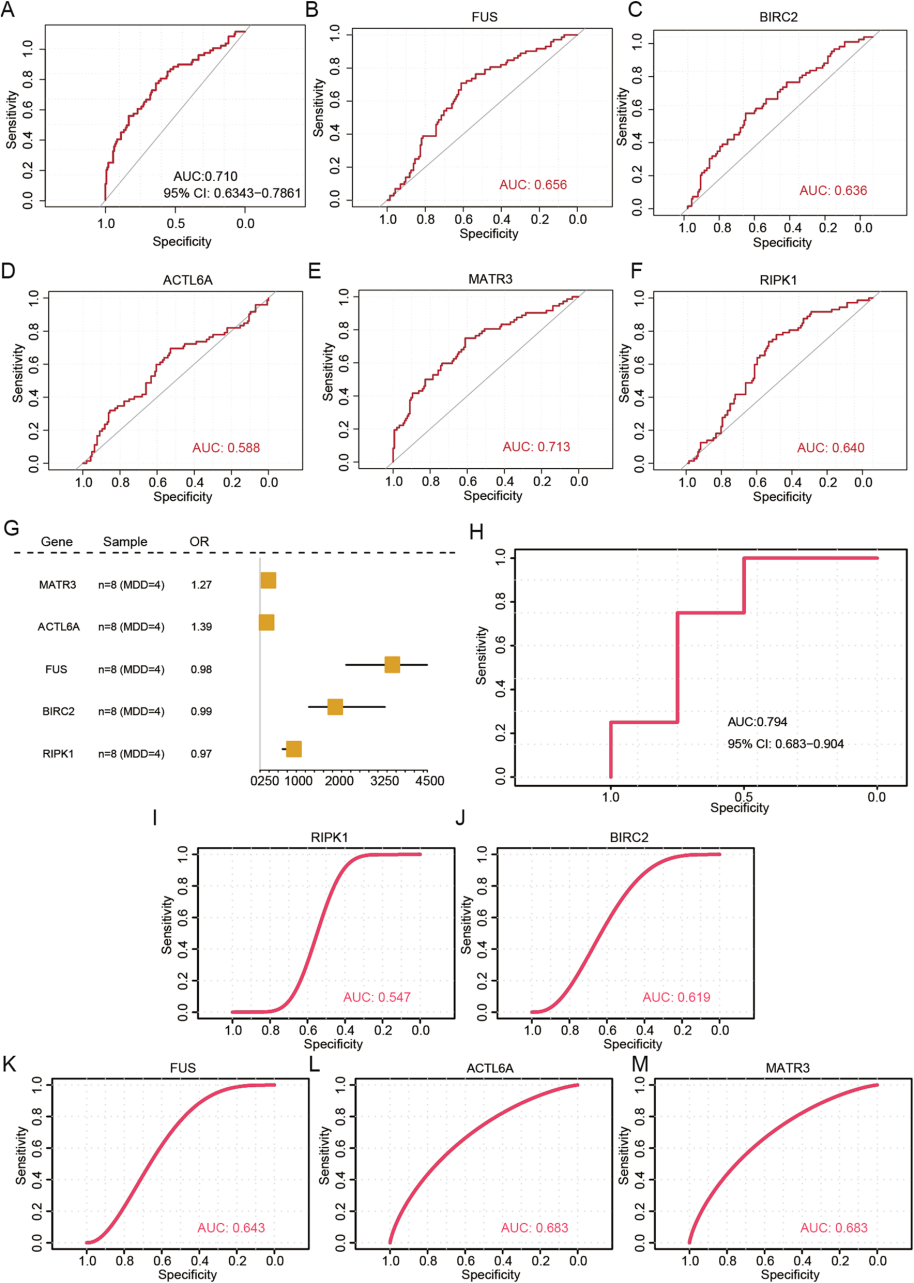
Diagnostic capacity assessment for candidate biomarkers. **A** Receiver operating characteristic (ROC) curves for the diagnostic power of biomarkers to differentiate major depressive disorder (MDD) from healthy controls in the training datasets. **B**-**F** Areas under the curve (AUCs) of the five biomarkers in the training dataset, respectively. **G** Forest plots for hub genes across the validation datasets (GSE190518). **H** ROC curves for the diagnostic ability of the five biomarkers to differentiate MDD from normal controls in the validation dataset. **I**-**M** AUCs of the five biomarkers in the validation dataset, respectively

Based on the training set for MDD, we developed a diagnostic prediction model using logistic regression and LASSO regression analyses. Five key genes were included in this model. AUC of 0.71 showed that the five key gene models had the potential to distinguish MDD from controls (Fig. 10A). In particular, the AUCs for *FUS*, *BIRC2*, *ACTL6A*, *MATR3*, and *RIPK1* were 0.656, 0.636, 0.588, 0.713, and 0.640, respectively, suggesting that these genes had good diagnostic ability (Fig. 10B-F). Furthermore, the diagnostic prediction model was evaluated using the independent validation set GSE190518. We visualized the expression data of the key genes using a forest plot (Fig. 10G). Further exploration of the diagnostic accuracy of the five key genes was conducted using the ROC curves. The AUCs for *MATR3*, *ACTL6A*, *FUS*, *BIRC2*, *RIPK1*, and the diagnostic model were 0.683, 0.683, 0.643, 0.619, 0.547, and 0.794, respectively (Fig. 10H-M). According to these findings, the five aforementioned biomarkers might be sensitive and specific to distinguish MDD samples from normal samples.

### Immune characteristics of the two subgroups

**Fig. 11:**
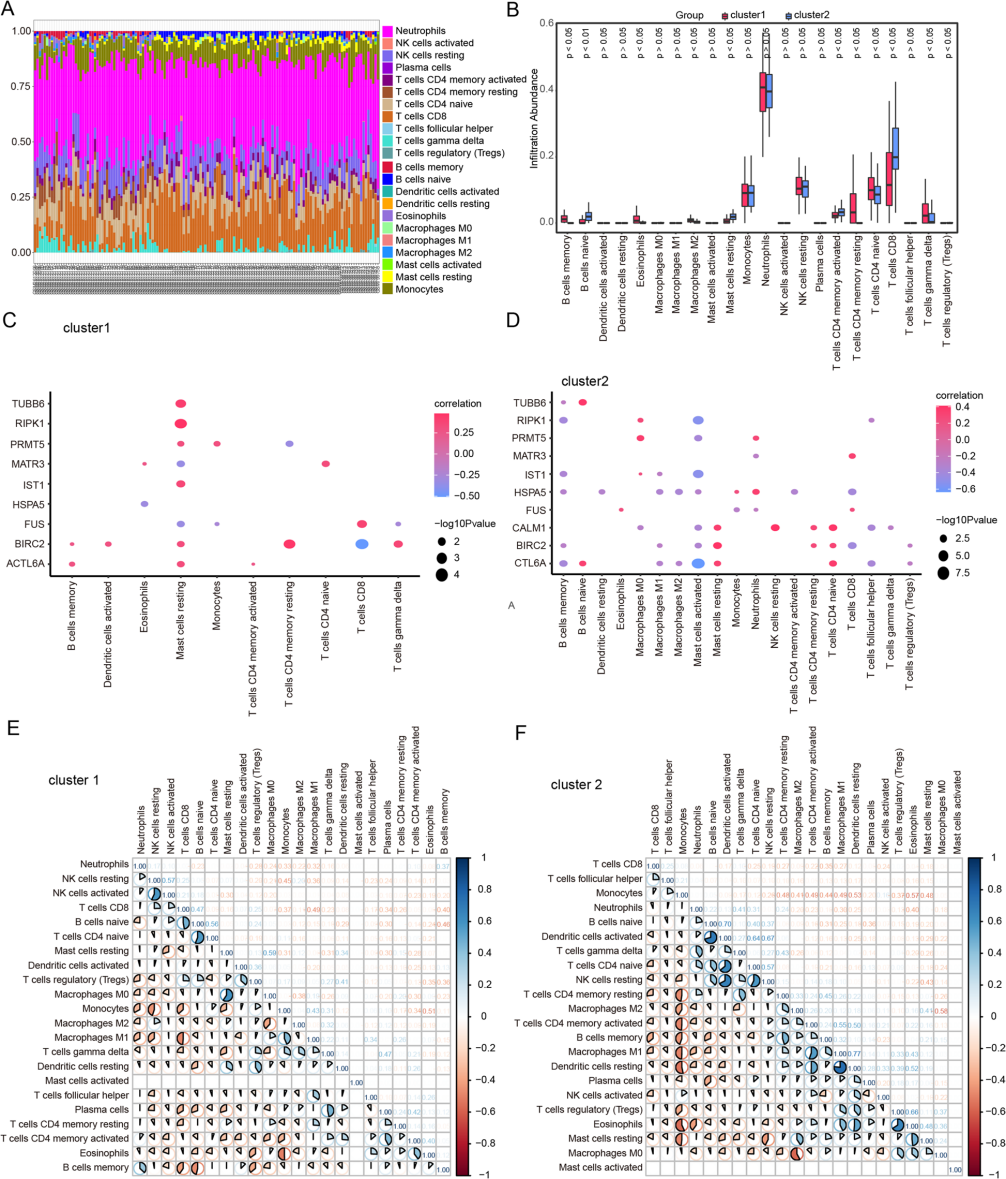
Immune infiltration analysis. **A** An analysis of the relative proportions of 22 types of immune infiltrating cells in clusters 1 and 2. **B** An analysis of 22 immune cells distributed between two clusters of major depressive disorder. The red color indicates the cluster 1 samples, and the blue color indicates the cluster 2 samples. **C**, **D** Correlation of the 10 hub genes with the immune cells in cluster 1 and cluster 2, respectively. **E** and **F** Matrix of correlation of immune cell proportions within cluster 1 and cluster 2

Using the CIBERSORT algorithm, we compared the relative abundance of 22 immune cells among the MDD clusters (Fig. 11A). We further investigated the differences in the proportions of 22 immune cells between cluster 1 and cluster 2. Cluster 1 showed significantly lower numbers of naïve B cells, resting mast cells, activated memory CD4 + T cells, and CD8 + T cells than cluster 2, while the numbers of memory B cells, M2 macrophages, naïve CD4 + T cells, resting memory CD4 + T cells, eosinophils, and gamma delta T cells were relatively higher in cluster 1 than cluster 2 (Fig. 11B). In addition, correlation analysis revealed that the top 10 hub genes in both clusters were differentially correlated with infiltrating immune cells. In both clusters, *ACTL6A* and *BIRC2* levels were strongly positively correlated with resting mast cells. *RIPK1* was positively correlated with resting mast cells in cluster 1 but negatively correlated with activated mast cells in cluster 2 (Fig. 11C, D).

Moreover, correlations between immune cell subpopulations showed significant differences between the two clusters. In cluster 1, there was a positive correlation between resting and activated NK cells, M0 macrophages, and resting mast cells (Fig. 11E). However, activated dendritic cells in cluster 2 were significantly positively correlated with naïve CD4 + T cells and resting NK cells (Fig. 11F).

### Construction of molecular subtypes based on biomarkers of MDD

**Fig. 12:**
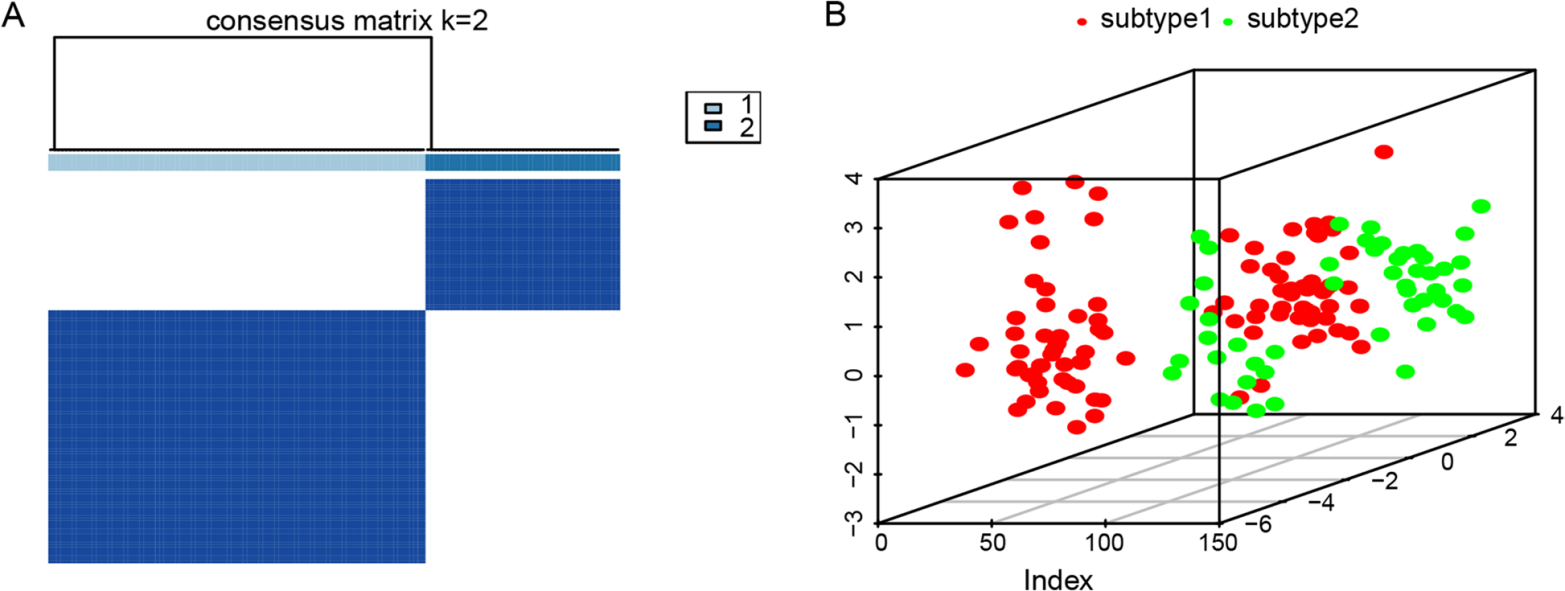
Molecular subtypes of major depressive disorder based on five biomarkers. **A** Clustering with K-means consensus (K = 2). **B** The PCA analysis showed the distribution pattern of subtype 1 and subtype 2

Based on the previously described five biomarker characteristics, MDD has again been divided into two distinct subtypes by consensus clustering using the R package “ConsensusClusterPlus” (Fig. 12A). Among them, subtype 1 contained 95 MDD samples and subtype 2 contained 49 MDD samples. As shown in Fig. 12B, PCA showed a difference in gene expression between the two subtypes.

### Identification of potential biomarkers for MDD

**Fig. 13:**
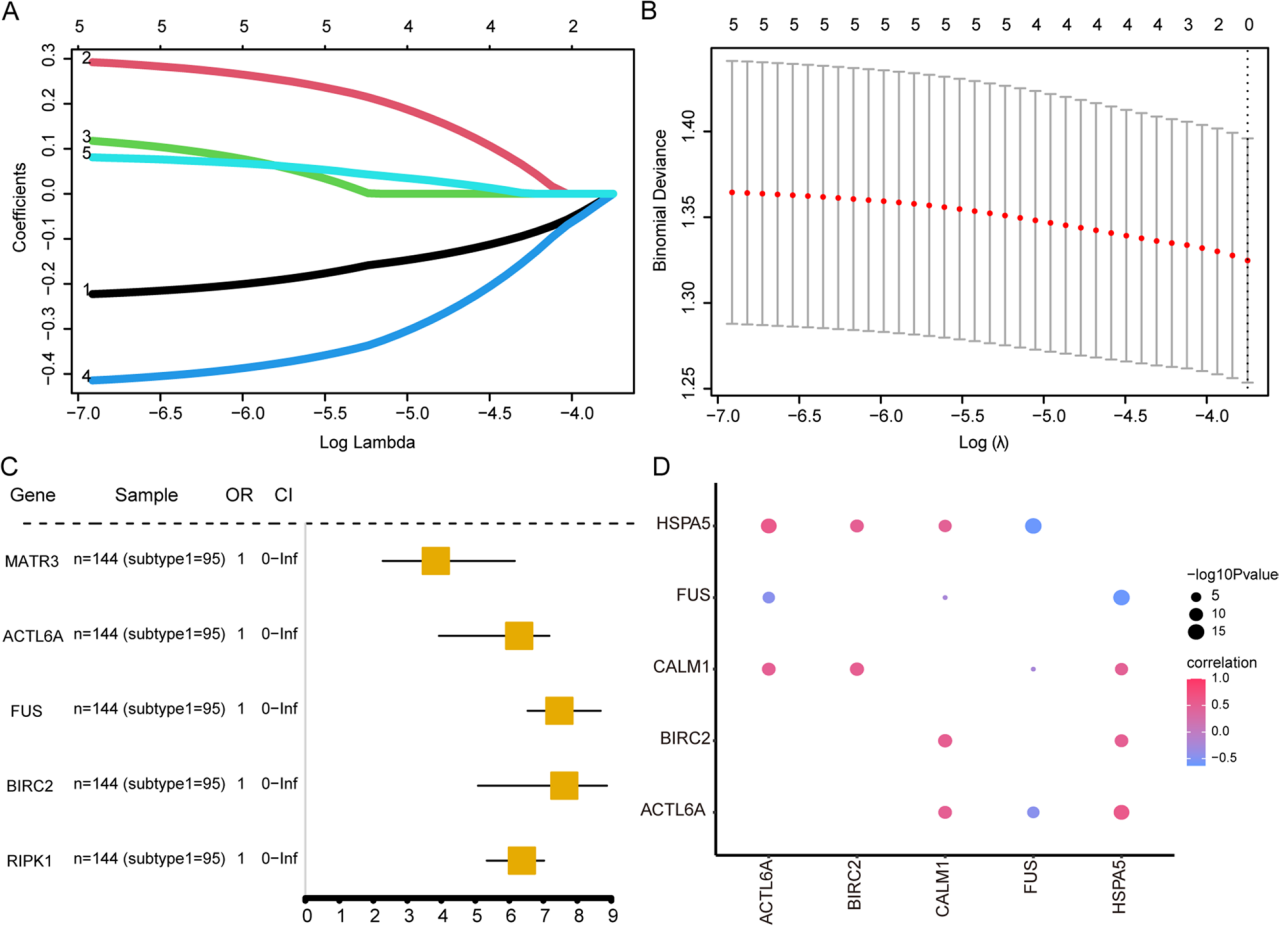
Correlation analysis of biomarkers based on major depressive disorder molecular subtypes. **A**-**B** Least absolute shrinkage and selection operator Cox regression model construction. **C** The forest plot shows the relationship between biomarkers and subtype 1 based on univariate logistic regression study results. **D** Correlation of the analyses between five biomarkers in two subtypes. Dot colors represent correlation coefficients and dot sizes represent *p*-values

To evaluate the influence of candidate biomarkers on patients with MDD subtypes, we used LASSO and univariate Cox regression analysis to screen for hub genes and evaluate the association between the five biomarkers and patients in two MDD clusters (Fig. 13A-C). Furthermore, correlation analysis of hub gene expression levels indicated that *ACTL6A*, *BIRC2*, and *CALM1* were significantly correlated with *HSPA5*, indicating that these genes tended to be co-expressed in MDD samples. The expression of *FUS* was negatively correlated with *HSPA5*, suggesting that *HSPA5* and FUS tended to be mutually exclusive in MDD samples (Fig. 13D).

**Fig. 14:**
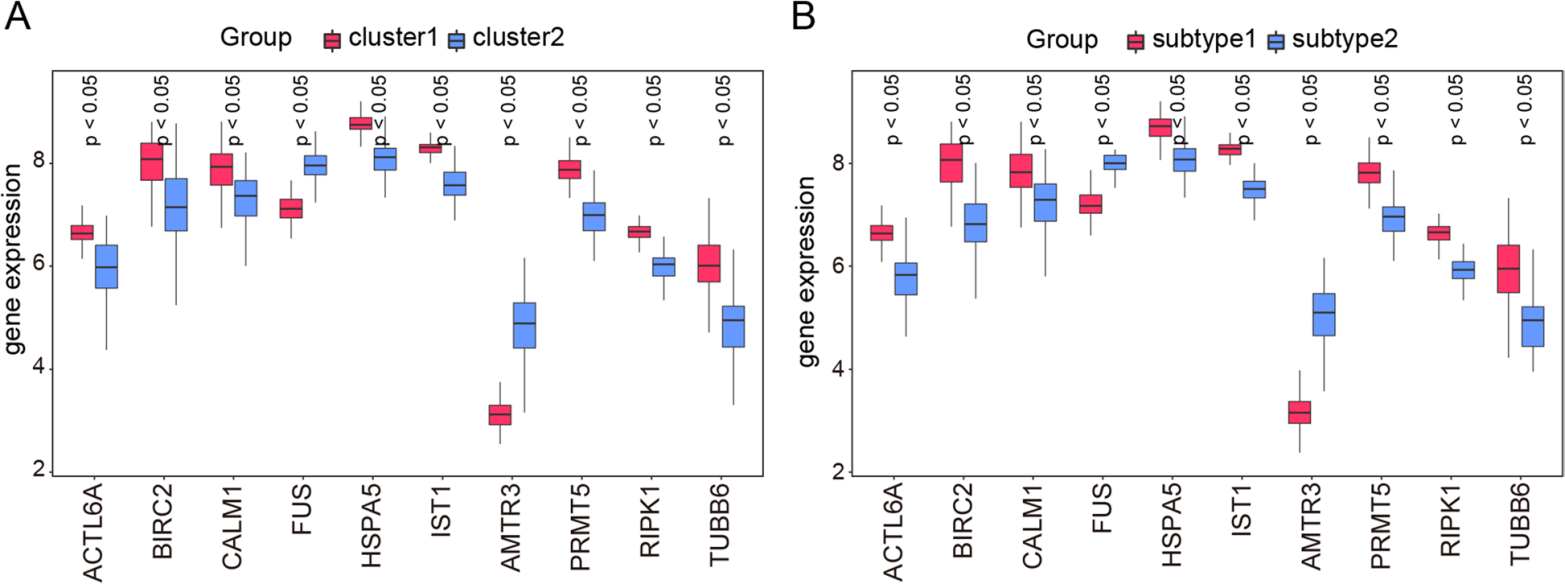
Analysis of the 10 hub genes expression levels. **A** The boxplot illustrates the differential levels of expression for the 10 hub genes in clusters 1 and 2. The red color denotes cluster 1 and blue color denotes cluster 2. **B** The boxplot illustrates the differential levels of expression for the 10 hub genes in subtype 1 and subtype 2. Red denotes subtype 1 and blue denotes subtype 2

To ensure consistency between the two types of clustering results, we created box plots to compare the expression levels of the 10 hub genes. Figure 14A, B shows the consistency between the first and second clustering results. Cluster 1 and subtype 1 showed higher expression levels of *ACTL6A*, *BIRC2*, and *RIPK1*, while cluster 1 and subtype 1 showed lower expression levels of *FUS* and *MATR3.* The results of the two types of consensus clustering analysis were highly coincident, indicating that the diagnostic marker genes had good distinguishing performance for MDD subtypes.

## Discussion

MDD is a highly heterogeneous disorder characterized by high levels of morbidity and mortality and is considered the most prevalent cause of disability worldwide [1, 2]. However, the pathophysiology of MDD remains unclear. Recently, high-throughput sequencing technologies have been used in many studies to elucidate the pathophysiological mechanisms of MDD and identify biomarkers for diagnosis [40, 41]. However, most of these studies have focused on differences between patients with MDD and healthy controls, while few have examined differences between MDD subtypes. Clinical practice cannot be guided by molecular subtypes due to the lack of subgroup classification, which hinders the implementation of precise treatment strategies for depression. Therefore, it is vital to broaden the study of MDD heterogeneity and identify new biomarkers to facilitate the early diagnosis and personalized treatment of MDD. Emerging evidence has revealed that mitophagy and immunity are related to the pathogenesis of depression [10, 15]. In light of this, we combined multiple bioinformatic approaches to identify mitophagy-related biomarkers of MDD and further investigate the function of immune cell infiltration in the disease. According to our results, two clusters of MDD presented different patterns of immune infiltration and gene signatures in unsupervised clustering analysis. Furthermore, we combined multiple functional enrichment analyses to reveal the potential mitophagy-related crosstalk involved in MDD. Finally, we identified five MRGs as key biomarkers of MDD, which can also be used to identify subtypes of MDD. Overall, this study identified a novel five-MRG gene signature that has good diagnostic performance and identified an association between MRGs and the immune microenvironment in MDD, which further reinforced the critical significance of mitophagy in diagnosing MDD and regulating the immune response [9, 10, 17, 42].

Mitophagy, a highly selective form of autophagy, utilizes multiple regulatory pathways to eliminate dysfunctional or redundant mitochondria in either ubiquitin-independent or ubiquitin-dependent manner [7]. According to GO analysis, 315 MR-DEGs are involved in biological processes and essential functions related to macroautophagy/autophagy and mitophagy, such as ubiquitin protein ligase binding. The ubiquitin–proteasome system (UPS) and autophagy pathways are critical for maintaining cellular homeostasis and are closely coordinated [43]. It is well known that *PINK1*/*Parkin* pathway-mediated mitophagy is the most extensively studied ubiquitin-dependent pathway for the clearance of damaged mitochondria [44]. Furthermore, aggregated studies have shown that polymorphisms in UPS-related genes are associated with MDD and antidepressant responses [45, 46]. The KEGG and DO analyses revealed that these MR-DEGs were related to neurodegenerative disorders, such as AD, PD, and ALS. These results are consistent with prior research describing the role of mitophagy in neurodegeneration [47, 48].

Five biomarkers (*MATR3, ACTL6A, FUS, BIRC2, and RIPK1*) were screened using four different algorithms, including WGCNA, LASSO, univariate regression analyses, and ROC curves. Subsequently, we reclassified MDD based on these biomarkers into two molecular subtypes, and our results showed that *ACTL6A*, *BIRC2*, and *RIPK1* were highly expressed in subtype 1, while *FUS* and *MATR3* were comparatively less expressed. Our study results further reinforce previous findings that the heterogeneity of depression may be related to different mitochondrial biological mechanisms [49].

MDD is a complex polygenic disease. Recent genetic studies suggest that single nucleotide polymorphisms (SNPs) in the *WFS1* and *CCKAR* genes may be associated with an increased risk of MDD [50, 51]. Mitochondria-related genes have also been shown to be involved in the development of MDD [52]. Our study further identified five mitophagy-related genes associated with MDD. Matrin 3 (*MATR3*), a protein in the nuclear matrix involved in DNA replication, apoptosis, and multiple RNA metabolism processes, is widely expressed in many tissues [53–55]. Several neurodegenerative diseases, including ALS and frontotemporal dementia, have been associated with *MATR3* mutations [54]. *FUS*, which shares structural and functional similarities with *MATR3*, is one of several RNA-binding proteins implicated in ALS [56]. A previous study has reported that *FUS* neurotoxicity is associated with the inhibition of autophagy and defective RNA metabolism [57]. Another study showed that *FUS* could be used as a candidate biomarker for MDD [58]. The ATP-dependent chromatin remodeling complex, *ACTL6A*, also known as *BAF53A* or *Arp4*, plays a key role in the development of progenitor cells, stem cells, and neuronal and hematopoietic cells [59]. ATP-dependent chromatin remodeling complexes are crucial for memory development and consolidation, neurodevelopment, and the etiology of depressive-like behavior [60, 61]. However, the role of *ACTL6A* in MDD has not been extensively investigated.

Several lines of evidence demonstrate that mitophagy and apoptosis are linked to the pathogenesis of MDD [9, 10, 62]. *BIRC2*, also known as the cellular inhibitor of apoptosis protein-1 (*cIAP1*), is an E3 ubiquitin-protein ligase that can promote the ubiquitination of *RIPK1*, thereby inhibiting *RIPK1*-mediated apoptosis and inflammatory responses [63–65]. Previous studies have revealed that *BIRC2* plays a critical molecular link between mitophagy and apoptosis [66]. Multiple pieces of evidence have confirmed that *RIPK1* is a key modulator of apoptosis, necroptosis, and neuroinflammation, and targeting *RIPK1* may inhibit multiple cell death pathways and alleviate neuroinflammation [67–69]. As a member of the receptor-interacting protein (*RIP*) kinase family, *RIPK1* has emerged as a target for intervention in inflammatory and neurodegenerative diseases [68, 69]. Furthermore, other *RIP* kinases, such as *PINK1 (PARK6)*, *PARK2 (Parkin)*, *PARK7*, and *LRRK2*, have also been reported to be involved in mitophagy [70, 71]. Interestingly, they are all Parkinson's disease-associated genes [71–73]. Among them, Parkin and *RIPK1/RIPK3* have been shown to be jointly involved in mediating necroptosis and inflammatory responses [74, 75]. In addition, in a study by Zeb S et al., fluoxetine exerts an antidepressive effect by indirectly inhibiting *RIPK1/RIPK3/MLKL*-mediated astrocytic necroptosis [76]. Overall, consistent with previous studies, the crosstalk between mitophagy, necroptosis, and inflammation may be closely related to the pathological mechanism of MDD [9, 62, 77]. *RIPK1* may play a critical molecular linkage role [76, 77].

Multiple studies have reported that MDD is linked to changes in innate and adaptive immune systems [14, 15, 78]. Compared to healthy controls, patients with depression have been shown to have increased neutrophil, monocyte, neutrophil/lymphocyte, CD4/CD8 cell-ratio, and T helper 17/T regulatory ratios [78]. Our results further reveal significant differences in immune infiltration between MDD and normal samples. Specifically, patients with MDD had a higher proportion of M1 macrophages, naïve B cells, resting mast cells, and activated memory CD4 + T cells than healthy controls, while the proportion of memory B cells and eosinophils was relatively lower. Consistent with previous studies, patients with MDD had elevated expression levels of M1 macrophages [79], mast cells [80], and memory T helper cells [81]. As reported by Singh et al., eosinophil count was slightly lower in both first-onset MDD and MDD with recurrent episodes [82]. However, it has been noted that studies regarding the overall number of circulating B cells in patients with MDD have returned conflicting results. Contrary to our results, previous studies have reported that the frequencies of naïve B cells, but not memory B cells, are reduced in severely depressed patients [83]. Interestingly, Yang et al. found decreased levels of B cells in patients with depression [84]. These inconsistencies might have stemmed from heterogeneous inclusion diagnostic criteria or the evaluation of varying severities of the disease. Overall, our results further confirm the relationship between immune dysregulation and MDD [14, 15].

Previous studies have shown that different subtypes of depression have different neurobiology, clinical courses, response to treatment, and immune characteristics [2, 85, 86]. Considering the heterogeneity of immune cells in MDD, two subgroups of patients with MDD with distinct immune cell patterns were identified using a consensus clustering algorithm. Compared to cluster 2, cluster 1 had higher expression of memory B cells, eosinophils, M2 macrophages, resting memory CD4 + T cells, naïve CD4 + T cells, and gamma delta T cells. The results indicated significant differences in immune patterns between the two clusters. Moreover, the important biomarkers *ACTL6A* and *BIRC2* were positively associated with resting mast cells in both clusters. In cluster 1, *RIPK1* expression was positively correlated with resting mast cells, while in cluster 2, it was negatively correlated with active mast cells. As antigen-presenting cells, mast cells contribute to inflammatory diseases through degranulation and the release of histamines and cytokines. There is substantial evidence linking mast cell involvement in inflammation pathways to depression [80, 87]. Furthermore, autophagy has been implicated in the degranulation of mast cells [88]. In the immune system, mitophagy has been shown to play a significant role [17, 42]. Psychiatric and neurodegenerative disorders are associated with malfunctioning mitophagy, mitochondria, and dysregulated activation of inflammasomes [9, 10, 18]. Based on these findings, several key mitophagy-related biomarkers, such as *BIRC2*, *ACTL6A*, and *RIPK1*, may be essential regulators of the immune status of patients with MDD and should be further investigated.

Although several bioinformatic approaches and statistical methodologies have been used to investigate diagnostic biomarkers, our study had several limitations. First, this study was a retrospective study; therefore, it lacked newly collected clinical samples and information. Second, the biological activities of the identified genes and their association with MDD are not fully understood. Due to the limited datasets in the database, the sample size of this study was insufficient, which may have led to bias. Due to the rarity and difficulty of acquiring normal human brain tissue samples, our study did not contain any controls consisting of brain tissue samples. As the research remained in the prediction stage, this study lacked sufficient experimental evidence to corroborate our prediction results. To improve our results, these data should be validated in vitro and in vivo.

## Conclusions

By combining multiple bioinformatic approaches, we identified five mitophagy-related biomarkers (*MATR3*, *ACTL6A*, *FUS*, *BIRC2*, and *RIPK1*) for MDD. Moreover, two mitophagy-based molecular subtypes and two distinct MDD-related clusters with diverse immune infiltrations in MDD were also identified, suggesting the importance of mitophagy in the diagnosis of MDD, regulating immune infiltration, and highlighting the biological heterogeneity of MDD. To the best of our knowledge, this study provides the first glimpse into the biological implications of MRGs and their relevance to immune cell infiltration in MDD. Identifying diagnostic indicators and molecular subtypes can lead to a deeper understanding of MDD’s molecular heterogeneity of MDD and help build accurate and individualized treatment options, thus reducing the burden of depression. However, further ex vivo and in vivo studies are necessary to confirm the accuracy of this study.

## Supplementary Information

**Additional file 1.**

## Data Availability

The datasets generated and/or analysed during the current study are available in the Gene Expression Omnibus database(https://www.ncbi.nlm.nih.gov/geo/); [GEO accession: GSE32280 (https://www.ncbi.nlm.nih.gov/geo/query/acc.cgi?acc=GSE32280), GSE98793(https://www.ncbi.nlm.nih.gov/geo/query/acc.cgi?acc=GSE98793), and GSE190518(https://www.ncbi.nlm.nih.gov/geo/query/acc.cgi?acc=GSE190518)].

## Abbreviations

MDD: Major depressive disorder
MRGs: Mitophagy-related genes
MR-DEGs: Mitophagy-related differentially expressed genes
DEGs: Differentially expressed genes
GEO: Gene Expression Omnibus
FC: Fold change
GO: Gene Ontology
BP: Biological processes
CC: Cellular component
MF: Molecular function
KEGG: Kyoto Encyclopedia of Genes and Genomes
GSEA: Gene set enrichment analysis
GSVA: Gene set variation analysis
DO: Disease Ontology
WGCNA: Weighted gene co-expression network analysis
PPI: Protein-protein interaction
STRING: Search Tool for the Retrieval of Interacting Genes
MCC: Maximal Clique Centrality
LASSO: Least absolute shrinkage and selection operator
ROC: Receiver operating characteristic
AUC: Area under the curve
