# Supplementary material for: Identification of mitophagy-related biomarkers and immune infiltration in major depressive disorder
Source: BMC Genomics. 2023 Apr 25;24:216. doi: 10.1186/s12864-023-09304-6 (PMC10131417; doi:10.1186/s12864-023-09304-6)
Supplement: Supplementary file 1 — Additional file 1. [file 12864_2023_9304_MOESM1_ESM.zip › Additional file 1/Supplementary Table S3 GO and KEGG enrichment analyses by GSEA.docx]

Supplementary Table S 3

**GO and KEGG enrichment analyses by GSEA**

| **Category** | **ID** | **Description** | **enrichmentScore** | **NES** | ***p*.adjust** |
| --- | --- | --- | --- | --- | --- |
| GOTERM_BP | GO:0097035 | regulation of membrane lipid distribution | -0.730844 | -2.528552 | 0.001145 |
| GOTERM_BP | GO:0034204 | lipid translocation | -0.739538 | -2.499991 | 0.001170 |
| GOTERM_BP | GO:0042908 | xenobiotic transport | -0.773742 | -2.495307 | 0.001218 |
| GOTERM_BP | GO:0044794 | positive regulation by host of viral process | 0.697042 | 2.038996 | 0.003436 |
| GOTERM_BP | GO:0036303 | lymph vessel morphogenesis | 0.513516 | 1.825158 | 0.003984 |
| GOTERM_CC | GO:0030119 | AP-type membrane coat adaptor complex | -0.776787 | -2.329239 | 0.001285 |
| GOTERM_CC | GO:0034045 | phagophore assembly site membrane | -0.805166 | -2.178984 | 0.001357 |
| GOTERM_CC | GO:0016469 | proton-transporting two-sector ATPase complex | -0.717518 | -2.135580 | 0.001302 |
| GOTERM_CC | GO:1902710 | GABA receptor complex | 0.646299 | 2.255439 | 0.004000 |
| GOTERM_CC | GO:1902711 | GABA-A receptor complex | 0.667242 | 2.223847 | 0.003891 |
| GOTERM_MF | GO:0016877 | ligase activity, forming carbon-sulfur bonds | -0.866601 | -2.782028 | 0.001217 |
| GOTERM_MF | GO:0016878 | acid-thiol ligase activity | -0.891615 | -2.715456 | 0.001274 |
| GOTERM_MF | GO:0140359 | ABC-type transporter activity | -0.803392 | -2.674768 | 0.001193 |
| GOTERM_MF | GO:0099095 | ligand-gated anion channel activity | 0.702882 | 2.309504 | 0.003774 |
| GOTERM_MF | GO:0016917 | GABA receptor activity | 0.650342 | 2.311471 | 0.003984 |
| KEGG_PATHWAY | hsa02010 | ABC transporters | -0.808865 | -2.626413 | 0.006631 |
| KEGG_PATHWAY | hsa04966 | Collecting duct acid secretion | -0.801394 | -2.380556 | 0.006631 |
| KEGG_PATHWAY | hsa04976 | Bile secretion | -0.647886 | -2.317522 | 0.006631 |
| KEGG_PATHWAY | hsa00071 | Fatty acid degradation | -0.699280 | -2.263988 | 0.001208 |
| KEGG_PATHWAY | hsa05033 | Nicotine addiction | 0.508268 | 2.051348 | 0.021579 |

Abbreviations: GO, Gene Ontology; BP, biological processes; CC, cellular component; MF, molecular function; KEGG, Kyoto Encyclopedia of Genes and Genomes; NES, Normalized Enrichment Score; GSEA, Gene set enrichment analysis.
