# Supplementary material for: Identification of mitophagy-related biomarkers and immune infiltration in major depressive disorder
Source: BMC Genomics. 2023 Apr 25;24:216. doi: 10.1186/s12864-023-09304-6 (PMC10131417; doi:10.1186/s12864-023-09304-6)
Supplement: Supplementary file 1 — Additional file 1. [file 12864_2023_9304_MOESM1_ESM.zip › Additional file 1/Supplementary Table S1 Detailed information about the three microarray datasets.docx]

Supplementary Table S 1

**Detailed information about the three microarray datasets.**

| **Category** | **GSE32280 [1]** | **GSE98793 [2]** | **GSE190518 [3]** |
| --- | --- | --- | --- |
| Organism | Homo sapiens | Homo sapiens | Homo sapiens |
| Experiment type | Expression profiling by array | Expression profiling by array | Expression profiling by array |
| Platforms | GPL570 | GPL570 | GPL20301 |
| Sample(number) | 192 | 32 | 8 |
| Sample of normal(number) | 64 | 8 | 4 |
| Sample of MDD (number) | 128 | 16 | 4 |

Abbreviations: MDD, Major depressive disorder

**References**

1. Yi Z, Li Z, Yu S, Yuan C, Hong W, Wang Z, Cui J, Shi T, Fang Y: **Blood-based gene expression profiles models for classification of subsyndromal symptomatic depression and major depressive disorder**. *PLoS One* 2012, **7**(2):e31283.

2. Leday GGR, Vértes PE, Richardson S, Greene JR, Regan T, Khan S, Henderson R, Freeman TC, Pariante CM, Harrison NA *et al*: **Replicable and Coupled Changes in Innate and Adaptive Immune Gene Expression in Two Case-Control Studies of Blood Microarrays in Major Depressive Disorder**. *Biol Psychiatry* 2018, **83**(1):70-80.

3. Zhang D, Ji Y, Chen X, Chen R, Wei Y, Peng Q, Lin J, Yin J, Li H, Cui L *et al*: **Peripheral Blood Circular RNAs as a Biomarker for Major Depressive Disorder and Prediction of Possible Pathways**. *Front Neurosci* 2022, **16**:844422.
