# Supplementary material for: Identification of mitophagy-related biomarkers and immune infiltration in major depressive disorder
Source: BMC Genomics. 2023 Apr 25;24:216. doi: 10.1186/s12864-023-09304-6 (PMC10131417; doi:10.1186/s12864-023-09304-6)
Supplement: Supplementary file 1 — Additional file 1. [file 12864_2023_9304_MOESM1_ESM.zip › Additional file 1/Supplementary Table S2 GO and KEGG pathway enrichment analysis of MR-DEGs in the most significant module.docx]

Supplementary Table S 2

**GO and KEGG pathway enrichment analysis of MR-DEGs in the most significant module**

| **Category** | **ID** | **Description** | **Count** | ***p*value** |
| --- | --- | --- | --- | --- |
| GOTERM_BP | GO:0016236 | macroautophagy | 30 | 3.18E-15 |
| GOTERM_BP | GO:1903008 | organelle disassembly | 18 | 1.10E-12 |
| GOTERM_BP | GO:0000422 | autophagy of mitochondrion | 13 | 1.76E-09 |
| GOTERM_BP | GO:0061726 | mitochondrion disassembly | 13 | 1.76E-09 |
| GOTERM_BP | GO:0022411 | cellular component disassembly | 28 | 2.33E-09 |
| GOTERM_CC | GO:0098798 | mitochondrial protein-containing complex | 30 | 1.77E-16 |
| GOTERM_CC | GO:0044391 | ribosomal subunit | 24 | 1.05E-15 |
| GOTERM_CC | GO:0005840 | ribosome | 25 | 3.82E-14 |
| GOTERM_CC | GO:0015934 | large ribosomal subunit | 17 | 2.42E-12 |
| GOTERM_CC | GO:1905369 | endopeptidase complex | 14 | 2.47E-11 |
| GOTERM_MF | GO:0003735 | structural constituent of ribosome | 24 | 5.94E-15 |
| GOTERM_MF | GO:0044389 | ubiquitin-like protein ligase binding | 21 | 1.19E-07 |
| GOTERM_MF | GO:0031625 | ubiquitin protein ligase binding | 18 | 3.60E-06 |
| GOTERM_MF | GO:0140597 | protein carrier chaperone | 5 | 9.68E-06 |
| GOTERM_MF | GO:0001094 | TFIID-class transcription factor complex binding | 4 | 1.57E-05 |
| KEGG_PATHWAY | hsa05020 | Prion disease | 31 | 1.49E-12 |
| KEGG_PATHWAY | hsa05012 | Parkinson disease | 30 | 4.25E-12 |
| KEGG_PATHWAY | hsa05022 | Pathways of neurodegeneration - multiple diseases | 39 | 4.78E-11 |
| KEGG_PATHWAY | hsa05010 | Alzheimer disease | 34 | 1.26E-10 |
| KEGG_PATHWAY | hsa05014 | Amyotrophic lateral sclerosis | 33 | 1.32E-10 |

Abbreviations: GO, Gene Ontology; BP, biological processes; CC, cellular component; MF, molecular function; KEGG, Kyoto Encyclopedia of Genes and Genomes; MR-DEGs, mitophagy-related differentially expressed genes.
