# Supplementary material for: Identification of mitophagy-related biomarkers and immune infiltration in major depressive disorder
Source: BMC Genomics. 2023 Apr 25;24:216. doi: 10.1186/s12864-023-09304-6 (PMC10131417; doi:10.1186/s12864-023-09304-6)
Supplement: Supplementary file 1 — Additional file 1. [file 12864_2023_9304_MOESM1_ESM.zip › Additional file 1/Supplementary Table S5-1 The top 20 most significantly enriched GO terms by GSVA.docx]

Supplementary Table S 5-1

**The top 20 most significantly enriched GO terms by GSVA**

| **Description** | **logFC** | **AveExpr** | **t** | ***P*.Value** | **adj.*P*.Val** | **B** |
| --- | --- | --- | --- | --- | --- | --- |
| go_re_entry_into_mitotic_cell_cycle | 0.81659 | -0.03204 | 5.04367 | 0.00000 | 0.00000 | 4.41545 |
| go_enzyme_directed_rrna_pseudouridine_synthesis | -1.15112 | 0.04796 | -10.23166 | 6.59E-19 | 6.09E-18 | 32.27506 |
| go_ribosomal_small_subunit_export_from_nucleus | -0.80977 | 0.04201 | -7.03141 | 7.05E-11 | 2.29E-10 | 14.01916 |
| go_box_c_d_snornp_assembly | -0.88284 | 0.06220 | -7.70272 | 1.78E-12 | 7.03E-12 | 17.63892 |
| go_protein_deneddylation | -0.65796 | 0.01924 | -6.51877 | 1.05E-09 | 2.98E-09 | 11.36624 |
| go_nucleotide_excision_repair_dna_damage_recognition | -0.76161 | 0.05109 | -8.95155 | 1.37E-15 | 8.01E-15 | 24.71415 |
| go_nucleotide_excision_repair_dna_duplex_unwinding | -0.68100 | 0.04562 | -7.42476 | 8.3E-12 | 3.03E-11 | 16.12186 |
| go_ribosomal_large_subunit_assembly | -0.91038 | 0.01821 | -10.39901 | 2.4E-19 | 2.38E-18 | 33.27829 |
| go_ribosomal_small_subunit_assembly | -1.00218 | 0.04564 | -12.20447 | 3.94E-24 | 8.4E-23 | 44.19959 |
| go_nuclear_transcribed_mrna_catabolic_process_nonsense_mediated_decay | -0.80090 | 0.03747 | -10.75415 | 2.77E-20 | 3.16E-19 | 35.41479 |
| go_spliceosomal_snrnp_assembly | -0.76283 | 0.01619 | -8.93756 | 1.49E-15 | 8.65E-15 | 24.63290 |
| go_rrna_modification | -0.71503 | 0.02594 | -8.69051 | 6.31E-15 | 3.42E-14 | 23.20516 |
| go_spliceosomal_snrnp_assembly | -0.76283 | 0.01619 | -8.93756 | 1.49E-15 | 8.65E-15 | 24.63290 |
| go_activation_of_mapkkk_activity | -0.78525 | 0.05937 | -10.22739 | 6.77E-19 | 6.23E-18 | 32.24948 |
| go_polysaccharide_catabolic_process | -0.70054 | 0.03906 | -9.90071 | 4.85E-18 | 4E-17 | 30.29937 |
| go_dna_catabolic_process_exonucleolytic | -1.49308 | 0.08158 | -17.27107 | 2.86E-37 | 1.96E-34 | 74.21439 |
| go_spliceosomal_conformational_changes_to_generate_catalytic_conformation | -1.26641 | 0.05930 | -12.19021 | 4.3E-24 | 9.07E-23 | 44.11303 |
| go_mrna_3_splice_site_recognition | -1.18909 | 0.07475 | -12.27753 | 2.52E-24 | 5.59E-23 | 44.64294 |
| go_autophagy_of_mitochondrion | -0.77249 | 0.03597 | -13.22426 | 7.79E-27 | 2.67E-25 | 50.37807 |
| go_mitophagy | -1.09788 | 0.05500 | -16.32891 | 6.7E-35 | 2.02E-32 | 68.80408 |

Abbreviations: GO, Gene Ontology; GSVA, Gene set enrichment analysis.
