# Supplementary material for: Identification of mitophagy-related biomarkers and immune infiltration in major depressive disorder
Source: BMC Genomics. 2023 Apr 25;24:216. doi: 10.1186/s12864-023-09304-6 (PMC10131417; doi:10.1186/s12864-023-09304-6)
Supplement: Supplementary file 1 — Additional file 1. [file 12864_2023_9304_MOESM1_ESM.zip › Additional file 1/Supplementary Table S4 The results of the Disease Ontology (DO) enrichment analysis.docx]

Supplementary Table S 4

**The results of the Disease Ontology (DO) enrichment analysis**

| **ID** | **Description** | **enrichmentScore** | **NES** | ***p*.adjust** |
| --- | --- | --- | --- | --- |
| DOID:10825 | essential hypertension | -0.480253312 | -1.815353804 | 2.89221E-05 |
| DOID:10211 | cholelithiasis | -0.63614594 | -1.93882525 | 0.000370096 |
| DOID:231 | motor neuron disease | 0.265036855 | 1.446569469 | 0.000519755 |
| DOID:1712 | aortic valve stenosis | -0.62008176 | -1.912719904 | 0.000635354 |
| DOID:332 | amyotrophic lateral sclerosis | 0.283887111 | 1.568582309 | 0.000740094 |
| DOID:205 | hyperostosis | 0.504822126 | 1.890048458 | 0.001426601 |
| DOID:8398 | osteoarthritis | -0.389931575 | -1.523305917 | 0.002467746 |
| DOID:0060262 | gallbladder disease | -0.560507734 | -1.804795163 | 0.002857809 |
| DOID:13580 | cholestasis | -0.49454732 | -1.710420782 | 0.002920422 |
| DOID:2355 | anemia | -0.371240563 | -1.474983865 | 0.003177128 |

Abbreviations: DO, Disease Ontology; NES, Normalized Enrichment Score.
