# Supplementary material for: Identification of mitophagy-related biomarkers and immune infiltration in major depressive disorder
Source: BMC Genomics. 2023 Apr 25;24:216. doi: 10.1186/s12864-023-09304-6 (PMC10131417; doi:10.1186/s12864-023-09304-6)
Supplement: Supplementary file 1 — Additional file 1. [file 12864_2023_9304_MOESM1_ESM.zip › Additional file 1/Supplementary Table S5-2 The top 20 most significantly enriched KEGG pathways by GSVA.docx]

Supplementary Table S 5-2

**The top 20 most significantly enriched KEGG pathways by GSVA**

| **Description** | **logFC** | **AveExpr** | **t** | ***P*.Value** | **adj.*P*.Val** | **B** |
| --- | --- | --- | --- | --- | --- | --- |
| kegg_linoleic_acid_metabolism | 0.47184 | -0.02156 | 6.20000 | 4.48E-09 | 9.68E-09 | 9.97463 |
| kegg_taste_transduction | 0.55373 | -0.02896 | 8.52717 | 9.82E-15 | 3.1E-14 | 22.77702 |
| kegg_glycosphingolipid_biosynthesis_lacto_and_neolacto_series | 0.26417 | -0.02103 | 3.63618 | 0.00037 | 0.00058 | -0.93393 |
| kegg_renin_angiotensin_system | 0.27998 | -0.00427 | 3.41560 | 0.00080 | 0.00124 | -1.65740 |
| kegg_glycosylphosphatidylinositol_gpi_anchor_biosynthesis | -0.41479 | 0.03188 | -4.72657 | 0.00000 | 0.00001 | 3.18194 |
| kegg_non_homologous_end_joining | -0.48778 | 0.02598 | -5.14318 | 7.68E-07 | 0.00000 | 4.97170 |
| kegg_riboflavin_metabolism | -0.51154 | 0.04088 | -5.85515 | 2.56E-08 | 5.29E-08 | 8.27441 |
| kegg_circadian_rhythm_mammal | -0.66245 | 0.04952 | -7.41371 | 6.37E-12 | 1.72E-11 | 16.39986 |
| kegg_n_glycan_biosynthesis | -0.70222 | 0.03200 | -9.86987 | 2.67E-18 | 1.1E-17 | 30.88977 |
| kegg_aminoacyl_trna_biosynthesis | -0.85446 | 0.03830 | -9.60020 | 1.42E-17 | 5.41E-17 | 29.23223 |
| kegg_pantothenate_and_coa_biosynthesis | -0.69549 | 0.04985 | -8.23794 | 5.46E-14 | 1.61E-13 | 21.08418 |
| kegg_snare_interactions_in_vesicular_transport | -0.78563 | 0.06259 | -11.92939 | 5.66E-24 | 5.02E-23 | 43.82369 |
| kegg_lysosome | -0.71981 | 0.04345 | -12.30778 | 5E-25 | 5.82E-24 | 46.22841 |
| kegg_sphingolipid_metabolism | -0.30774 | 0.00163 | -4.19292 | 0.00005 | 0.00007 | 1.05956 |
| kegg_nicotinate_and_nicotinamide_metabolism | -0.38449 | 0.01408 | -5.26629 | 4.35E-07 | 8.34E-07 | 5.52149 |
| kegg_nicotinate_and_nicotinamide_metabolism | -0.38449 | 0.01408 | -5.26629 | 4.35E-07 | 8.34E-07 | 5.52149 |
| kegg_glycerolipid_metabolism | -0.39463 | 0.01861 | -7.09348 | 3.8E-11 | 9.96E-11 | 14.64430 |
| kegg_glycerophospholipid_metabolism | -0.42694 | 0.01838 | -8.09797 | 1.24E-13 | 3.62E-13 | 20.27346 |
| kegg_glycosphingolipid_biosynthesis_ganglio_series | -0.52839 | 0.01515 | -7.08715 | 3.94E-11 | 1.02E-10 | 14.60999 |
| kegg_other_glycan_degradation | -0.76278 | 0.05378 | -9.33735 | 7.22E-17 | 2.63E-16 | 27.62876 |
| kegg_glycosaminoglycan_degradation | -0.41443 | -0.00615 | -5.06612 | 0.00000 | 0.00000 | 4.63229 |

Abbreviations: KEGG, Kyoto Encyclopedia of Genes and Genomes; GSVA, Gene set enrichment analysis.
